# Transport physics‐informed reinforcement learning agents deployed in standalone infusion pumps for managing multidrug delivery in critical care

**DOI:** 10.1002/btm2.70013

**Published:** 2025-03-18

**Authors:** V. Chandran Suja, A. L. H. S. Detry, N. M. Sims, D. E. Arney, S. Mitragotri, R. A. Peterfreund

**Affiliations:** ^1^ School of Engineering and Applied Sciences Harvard University Boston Massachusetts USA; ^2^ Wyss Institute for Biologically Inspired Engineering Cambridge Massachusetts USA; ^3^ Department of Anesthesia, Critical Care and Pain Medicine Massachusetts General Hospital Boston Massachusetts USA; ^4^ Department of Chemical, Materials and Industrial Production Engineering University of Naples Federico II Naples Italy

**Keywords:** advection diffusion equation, drug delivery, physics‐informed machine learning, reinforcement learning

## Abstract

Managing delivery of complex multidrug infusions in anesthesia and critical care presents a significant clinical challenge. Current practices relying on manual control of infusion pumps often result in unpredictable drug delivery profiles and dosing errors—key issues highlighted by the United States Food and Drug Administration (FDA). To address these issues, we introduce the SMART (synchronized‐pump management algorithms for reliable therapies) framework, a novel approach that leverages low Reynolds number drug transport physics and machine learning to accurately manage multidrug infusions in real‐time. SMART is activated based on the Shafer number (Sh), a novel non‐dimensional number that quantifies the relative magnitude of a drug's therapeutic action timescale to its transport timescale within infusion manifolds. SMART is useful when Sh<1, where drug transport becomes the rate limiting step in achieving the desired therapeutic effects. When activated, SMART monitors multidrug concentrations within infusion manifolds and leverages this information to perform end‐to‐end management of drug delivery using an ensemble of deterministic and deep reinforcement learning (RL) decision networks. Notably, SMART RL networks employ differentially sampled split buffer architecture that accelerates learning and improves performance by seamlessly combining deterministic predictions with RL experience during training. SMART deployed in standalone infusion pumps under simulated clinical conditions outperformed state‐of‐the‐art manual control protocols. This framework has the potential to revolutionize critical care by enhancing accuracy of medication delivery and reducing cognitive workloads. Beyond critical care, the ability to accurately manage multi‐liquid delivery via complex manifolds will have important bearings for manufacturing and process control.

**Translational Impact Statement**

The ability to accurately deliver multi‐drug infusions without transport delays is of particular interest in critical care scenarios, as highlighted by recent FDA notices. We address this need by introducing a clinically translatable framework combining analytical and deep reinforcement learning (RL) decision networks to automatically manage drug delivery end‐to‐end in scenarios benefiting from intervention identified through a new dimensionless number. When deployed in standalone infusion pumps, this technology significantly outperformed manual intervention techniques by reducing delivery delays in simulated critical care scenarios.

## 1 INTRODUCTION

Mitigating medication errors has been a high priority in anesthesia and critical care since the publication of “To Err Is Human” by the National Academy of Medicine in 1999.^1^ Manually managed multidrug infusions utilizing medical grade pumps, particularly at low infusion rates, is a major source of medication errors resulting from unpredictable delivery profiles and delivery delays.^2^, ^3^, ^4^ As anesthesia and critical care procedures continue to become more sophisticated and prevalent, particularly with advances in cardiac surgeries,^5^ organ transplants,^6^ and neonatal care,^7^, ^8^ significant innovation is needed to reduce medication errors and facilitate front‐line caregivers in delivering ever‐more nuanced critical care. Such innovations will also help in reducing clinical cognitive workloads and expediting clinical decision‐making, particularly pressing needs for health care systems that are strained with very low clinician–patient ratios.^9^, ^10^ Major strides have been made to mitigate medication errors through innovations such as Barcode Medication Administration (BCMA),^11^, ^12^ infusion pumps with in‐build medication databases^13^ and infusion guard‐railing.^14^ However, the efforts so far have overlooked one of the major drivers for medication errors, the obscured transport of drugs in infusion manifolds.^15^, ^16^, ^17^, ^18^

**FIGURE 1:**
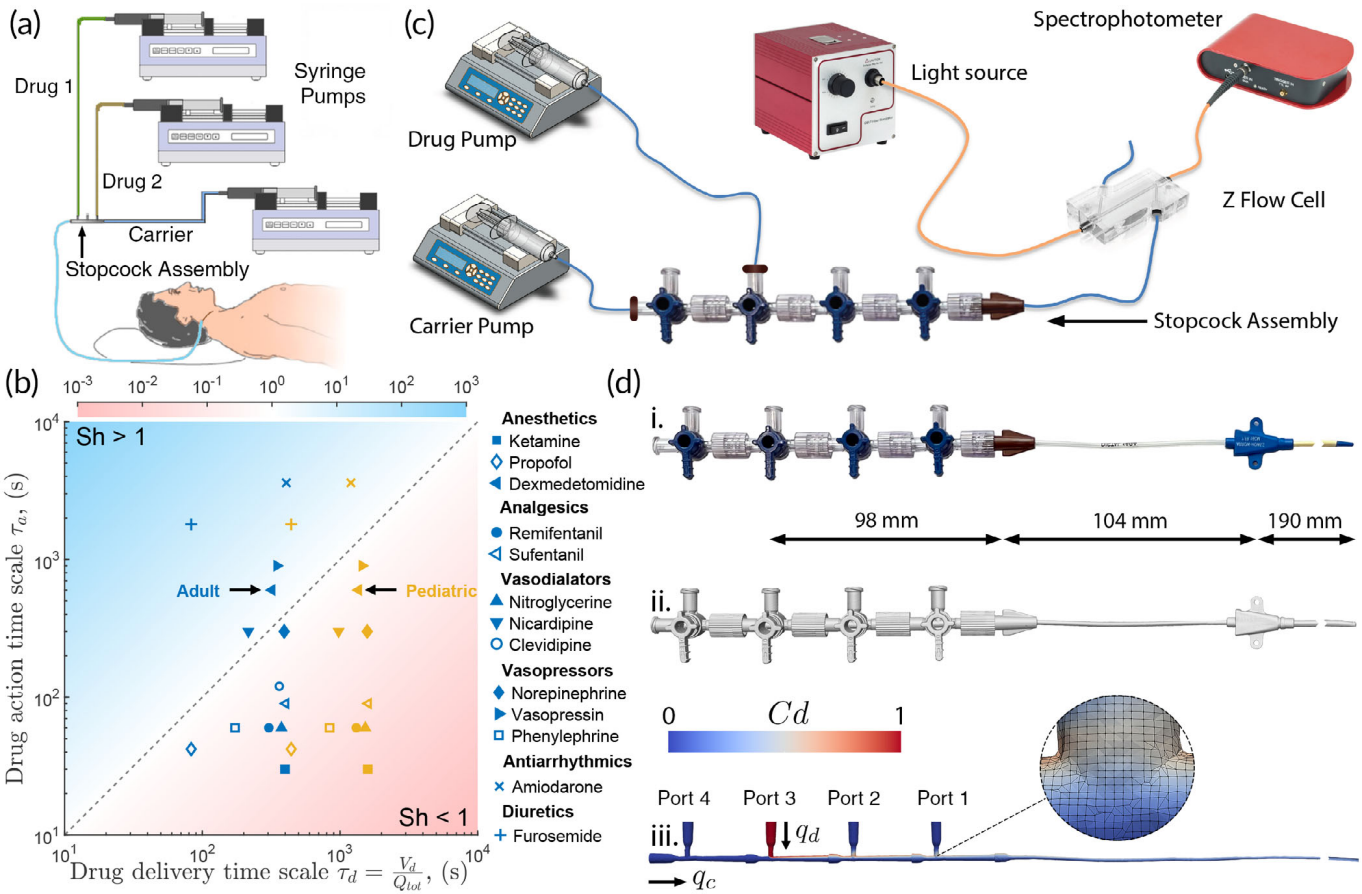
(a) Schematic showing a multi‐drug infusion with a central venous catheter in a critical care setting. (b) Shafer number of common drugs administered in critical care. Aligning with conventional wisdom, most of the tightly titrated drugs that benefit from SMART have low Shafer numbers (*Sh* < 1). Drug administered under ‘adult’ conditions are shown in blue, while ‘pediatric’ conditions are shown in orange. Color bar indicates values of the Shafer number. See Table S1 for additional details. (c) Bench top spectrophotometer setup used to experimentally measure multi drug infusion kinetics. (d) Geometry preparation for fully resolved numerical simulations. The entire infusion manifold assembly comprising four stopcocks and a triple lumen catheter (i.), was microCT scanned (ii.), and the internal fluid volume was extracted with ORS DragonFly (iii.). The fluid volume was meshed into hexahedral volumes using OpenFOAM SnappyHexMex (inset). *C*_d_ is the drug concentration, and ports for drug infusion are numbered from the tip of the catheter. $q_{d}$ and $q_{c}$ are the drug and carrier volumetric flow rates respectively.

In operating rooms and intensive care units, multiple life‐critical medications are administered by pumps via infusion manifolds comprising stopcocks and catheters (Figure 1a). The drugs and carrier fluid infused by the pumps mix and move hydrodynamically coupled along the infusion manifold, before being delivered into the bloodstream at the tip of a catheter. Manually managing multidrug infusions is a cognitively demanding task as multiple drugs may have to be started, stopped, or dose adjusted in short time frames, while tightly controlling the total fluid delivered to the patient (Figure S1). Accurate management of these dynamic high‐stakes infusions are further complicated by three factors affecting transport—concentrated drugs flowing at low flow rates (0.1–3.0 mL/h), hydrodynamic coupling, and the dead volume.^3^, ^19^ Hydrodynamic coupling refers to the phenomenon where the flow of drugs influences each other within the infusion manifold, mutually affecting their delivery kinetics. As a result, addition or removal of a drug transiently affects the delivery of all the drugs in the system. The dead volume refers to the total fluid volume between the drug's entry point into the manifold and its delivery point. This dead volume, often as large as 10 times the drug volume infused in an hour, can introduce significant delays in achieving a desired dose‐rate, leading to a mismatch between clinical intention and actual drug delivery profiles.^4^, ^20^ Lacking specific knowledge of real‐time drug levels within the dead volume, clinicians are currently forced to use educated guesses to manually adjust drug flow rates on multiple infusion pumps to overcome drug delivery delays (Figure S2), possibly leading to suboptimal outcomes often including peri‐intravenous hemorrhages and severe oscillations in vital parameters such as blood pressure or cardiac contractility.^2^, ^3^

We report here a clinically translatable framework for tackling this problem that leverages accurate reduced‐order drug transport modeling and real‐time synchronized control of infusion pumps. This computationally efficient framework called SMART (synchronized‐pump management algorithms for reliable therapies) can be deployed within stand‐alone infusion pumps and opens a scalable solution to a multitude of problems in multidrug delivery while helping to decrease medication errors and clinical workloads. Previous efforts reported for deterministic^18^, ^21^, ^22^ or machine learning‐based control^23^, ^24^, ^25^ of infusion pumps are focused on managing physiological variables for single‐drug infusions. SMART, on the other hand, is designed to handle coupled transport of multiple drugs. This ability to manage multidrug transport will aid in translating previously reported single‐drug physiological control frameworks to anesthesia and critical care scenarios.

The rest of the article is organized as follows. First, we establish a non‐dimensional number, which we term the Shafer number, to objectively identify situations where drug transport confounds clinical expectations and leads to delivery delays and dosing errors. Subsequently, we report the development of a reduced‐order transport model leveraging the Gill–Sankarasubramanian^26^ approximation to the advection–diffusion equation, which we validate utilizing a benchtop experimental platform and through fully resolved numerical simulations. We then detail a deterministic framework for real‐time control of infusion pumps to rapidly and accurately attain clinical objectives. Subsequently, we report an OpenAI gym‐compatible ‘infusion gym’ and train drug‐transport‐informed reinforcement learning agents that can execute end‐to‐end synchronized control of multi‐drug infusions exceeding the performance of deterministic modeling. Finally, we integrate this computationally efficient SMART framework within stand‐alone infusion pumps, across both syringe pumps and peristaltic pumps, and demonstrate its capability to reduce delivery delays and dosing errors. We conclude the article by discussing the broad implications of the SMART framework, including for manufacturing and process control.

## 2 RESULTS AND DISCUSSION

### 2.1 The Shafer number

We first establish a non‐dimensional number, which will serve as a unified and an objective way to identify clinical scenarios benefitting from active management of drug infusions. This non‐dimensional number physically quantifies the relative importance of two time scales that ultimately dictates how quickly an infused drug displays its therapeutic effect. The first timescale, $\tau_{a}$, corresponds to the time it takes for a drug to act on cellular targets and elicit the appropriate biochemical response *after* it enters the body (Figure S3a). This timescale is well known for many drugs (Table S1), and among other things depend on the physicochemical characteristics and the pharmacokinetics of the drug. Another timescale enters the problem solely due to the advective transport of the drug within infusion manifolds (Figure S3b). For an infusion manifold with a dead volume $V_{\text{dead}}$ and total drug flow rate $Q_{\mathrm{tot}}$, the timescale for the drug to reach the catheter tip (delivery point) can be shown to scale as, $\tau_{p}=V_{\text{dead}}/\left(Q_{\mathrm{tot}}\right)$ (Figure S3). We propose to call the ratio of these two time scales as the Shafer number, $\mathrm{Sh}=\tau_{a}Q_{\mathrm{tot}}/V_{\text{dead}}$, in honor of Steven L. Shafer, M.D., who pioneered significant advances in infusion pump mediated drug delivery.^27^, ^28^

Shafer number (Sh) of common drugs administered in critical care are shown in Figure 1b. Notably drugs requiring minimal delivery management such as amiodarone and furosemide (Sh>1) are delineated from those that are tightly managed in the clinic such as clevidipine and ketamine (Sh<1). At high Shafer numbers (Sh>1), drug transport kinetics by definition is not rate limiting. Consequently, there is no benefit in implementing techniques to improve delivery kinetics in this regime, including the proposed SMART method. On the other hand, at low Shafer numbers, drug transport kinetics through the catheter effectively dictates the time taken for an infused drug to display its therapeutic effect. It is precisely these conditions that are most prone to delivery delays, and consequently benefit from active management of drug infusions in the clinic. By simply computing the Shafer number, we now have a physically grounded metric to identify scenarios that require drug delivery management, without relying on clinical experience.

From the definition of the Shafer number, we can gain further insights into three relevant situations that benefit from drug delivery management. First, infusions with fast acting drugs, that is, low $\tau_{a}$. Many drugs common in anesthesia and critical care settings including several vasopressors, vasodilators, analgesics, and anesthetics are fast acting (Table S1). Second, infusions with low total drug infusion rate, that is, low $Q_{\mathrm{tot}}$. Low flow rate infusions are common in pediatric and neonatal intensive care units. Third, infusions through geometries with large dead volume, that is, high $V_{\text{dead}}$. Legacy stopcock assemblies common in both adult and pediatric intensive care settings have large dead volumes. Taken together, these three scenarios account for a significant fraction of all anesthetizing and intensive care situations (Figure 1b). Motivated by this broad need, we developed SMART to improve multi‐drug infusions at low Shafer numbers.

### 2.2 Framework for developing and testing SMART

We established a high‐fidelity framework comprising an experimental platform and OpenFOAM‐based fully resolved numerical simulations for accelerated development and testing of SMART (Figure 1). The experimental platform mimics a bedside central venous catheter setup (Figure 1a) equipped with a spectrometer to obtain time‐resolved drug concentration measurements (Figure 1c). The tip of the catheter is interfaced with a low‐volume Z‐flow cell to provide a large optical path and enhance the signal‐to‐noise ratio of spectroscopic measurements while minimizing additional dead volume. Food‐grade dyes erioglaucine and tartrazine, which have distinct spectral absorption signatures (Figure S4), are used as model drugs. During a typical experiment, computational platforms running SMART are interfaced with pumps loaded with saline and dye(s) of known concentration, and drug delivery performance is evaluated by measuring the temporal evolution of dye concentrations at the catheter tip. For accelerating the development and orthogonal validation of SMART, we performed fully resolved numerical simulation using OpenFOAM. A catheter–stopcock assembly identical to that used in the experiments (Figure 1d.i) was microCT scanned (Figure 1d.ii) and processed in ORS DragonFly to extract the internal fluid volume (Figure 1d.iii). The volume was meshed using the snappy hex mesh, and drug transport was resolved with adaptive time stepping employing a custom advection–diffusion equation solver based on the native IcoFoam solver (see Section 4). A mesh independence study was performed prior to using the model for evaluating SMART (Figure S5).

### 2.3 Computationally efficient reduced order model for drug transport

Deploying SMART for real‐time control of drug infusions in clinical settings requires computationally efficient algorithms that can be executed in small‐footprint hardware. The key to accomplishing this goal rests on developing accurate reduced‐order models for accurately tracking drug transport within the catheter.

The generalized 3D advection diffusion equation (ADE) that governs drug transport, can be computationally simplified by neglecting radial and azimuthal variations, and focusing solely on the axial evolution of the drug concentration. We realize such an approximation following Gill and Sankarasubramanian,^26^ which holds for fully developed Poiseuille flows with constant and isotropic species diffusivity (D). Unlike the Taylor diffusion approximation,^29^ which is only accurate for time scales $\tau =\mathrm{Dt}/a^{2}⪆0.2$, the Gill–Sankarasubramanian approximation is accurate at arbitrarily small time scales (Figure S6).^26^ Here t is time and a is the radial extent of the geometry. Variations in the radial extent of the stopcock–catheter assembly are captured through a network model^30^ with piece‐wise continuous cylindrical units with matched outlet–inlet boundary conditions. These assumptions are valid for modeling drug transport through stopcock–catheter assemblies for conditions encountered in critical care settings (Figure S7).

**FIGURE 2:**
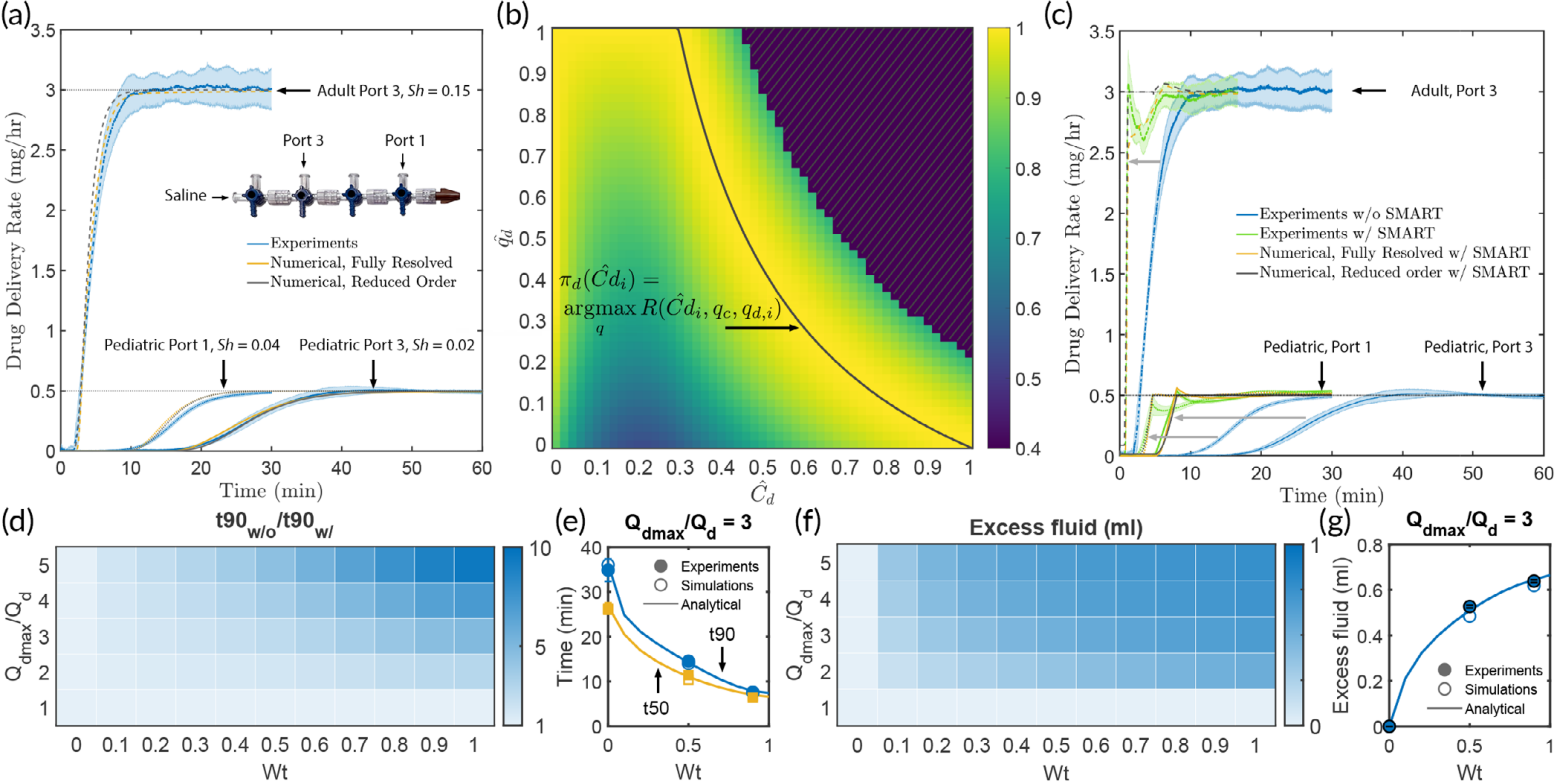
Drug infusion with synchronized‐pump manipulation using the deterministic framework. (a) Baseline drug infusion kinetics for three cases without SMART (see Figure S9 for drug and carrier infusion rates). The indicated Shafer numbers are calculated for a drug behaving like propofol ($\tau_{a}=40\;s$). (b) A heatmap showing state‐action‐values of the deterministic framework in the $\hat{q_{d}}-\hat{C_{d}}$ plane. The solid line shows the effective policy $\pi_{d}\left(\hat{{C_{d}}_{i}}\right)$ executed by the deterministic framework. (c) SMART with deterministic algorithms improves drug delivery kinetics by dramatically reducing the time required to reach the steady state. For all cases, $Q_{\text{dmax}}/Q_{d}=3$. (d) A numerical parameter sweep showing the impact of maximum flow rate factor ($Q_{\text{dmax}}/Q_{d}$) and excess fluid parameter ($W_{t}$) on the relative improvement in time to reach 90% of set drug delivery rate ($t90_{w/o}/t90_{w/\text{SMART}}$). No improvement in delivery kinetics is physically possible when $Q_{\text{dmax}}/Q_{d}=0$ or $W_{t}=1$. (e) The results of the parameter sweep in d. are corroborated by experiments and fully resolved numerical simulations. Both t90 and t50 are shown for $Q_{\text{dmax}}/Q_{d}=3$. (f) A numerical parameter sweep showing the impact of maximum flow rate factor ($Q_{\text{dmax}}/Q_{d}$) and excess fluid parameter ($W_{t}$) on the excess fluid volume dispensed to reach 90% of set drug delivery rate. (g) The results of the parameter sweep in (f) are corroborated by experiments and fully resolved numerical simulations. Data shown for $Q_{\text{dmax}}/Q_{d}=3$.

The reduced order model is solved numerically using the second order accurate Crank‐Nicholson finite difference scheme.^31^ Numerical oscillations are minimized by employing the Backward‐Euler scheme to evolve the first step,^32^ where sharp gradients in drug concentration are present. Sparse matrix implementation of the schemes is adopted to significantly enhance the computational and memory management efficiency. Overall the adopted reduced order model, when compared to fully resolved numerical simulation, provides a 10,000‐fold enhancement in computational time on a per‐core physical basis (Figure S8), while providing accurate resolution of drug delivery profiles across a range of infusion conditions prevalent in anesthesia and critical care (Figures 2a and S9). These profiles also reveal that the extent of delivery delays scale with the Sh number, with situations common in pediatric settings requiring over 30 min to reach the desired delivery rates at the catheter tip. Minimizing drug delivery delays is a central goal of the SMART framework, which we accomplish via an ensemble of deterministic and reinforcement learning based approaches.

### 2.4 Deterministic policy for rapid drug delivery

At its core, the developed deterministic policy chooses actions that minimize the error between the expected and actual drug delivery rates at a given instant of time. The possible actions are independently varying the instantaneous drug pump infusion rate $q_{d,i}$ and the carrier infusion rate $q_{c}$ for each state defined by the drug concentration ${c_{d}}_{i}$ at the catheter tip. The reward $R\left({\hat{C_{d}}}_{i},q_{c},q_{d,i}\right)$ for an action is simply defined as the negative of error in drug delivery as (see Appendix S1),

(1) $$R\left(\hat{{C_{d}}_{i}},q_{c},q_{d,i}\right)=-\exp\left(\left(Q_{c}+\sum_{n=1}^{i}Q_{d,i}\right)-\left(q_{c}+\sum_{n=1}^{i}q_{d,i}\right)\hat{{C_{d}}_{i}}\right)-{\left(1+{c_{d}}_{i}\right)}^{2}\left(1-W_{t}\right){\left(Q_{c}-q_{c}\right)}^{2}-{\left(1-{c_{d}}_{i}\right)}^{2}W_{t}{\left(Q_{\text{cmax}}-q_{c}\right)}^{2}$$

Subject to,

(2) $$0\leq q_{c}\leq Q_{\text{cmax}}$$

(3) $$\begin{array}{c} 0\leq q_{d,i}\leq Q_{\text{dmax},i} \end{array}$$

(4) $$\begin{array}{c} \frac{q_{d,i}}{q_{d,i}+q_{c}}\leq \left(1+\kappa \right)\frac{Q_{d,i}}{Q_{d,i}+Q_{c}} \end{array}$$

(5) $$\begin{array}{c} \frac{q_{d,i}}{q_{d,i}+q_{c}}\geq \left(1-\kappa \right)\frac{Q_{d,i}}{Q_{d,i}+Q_{c}} \end{array}$$

(6) $$\begin{array}{c} \left(q_{c}+\sum_{n=1}^{i}q_{d,i}\right)\hat{{C_{d}}_{i}}\leq \beta \left(Q_{c}+\sum_{n=1}^{i}Q_{d,i}\right) \end{array}$$

Here $W_{t}$ is a weighting parameter that linearly controls the amount of excess carrier fluid, $Q_{c}$ and $Q_{d}$ are respectively the set carrier and drug flow rates, $Q_{\text{cmax}}$ and $Q_{\text{dmax}}$ are respectively the maximum permissible carrier and drug flow rates, while κ and β are coefficients that respectively limit the drug concentration fluctuations and the drug delivery overshoots. Physically, the first term in Equation (1) expresses the difference between desired and actual drug delivery rate at the catheter tip, while the last two terms control the excess carrier fluid based on $W_{t}$. The first two constraints enforce the range of syringe flow rates that are deemed clinically safe (or within hardware constraints), the next two constraints limit fluctuation in concentration at the inlet relative to the desired steady state value, and the last constraint places an upper bound on the permissible overshoot in drug concentration at the outlet. For improved stability during nonlinear optimization and for reinforcement learning, in practice we solve a non‐dimensional version of Equation (1) where the drug concentration is normalized by the steady state value ($\hat{{C_{d}}_{i}}={c_{d}}_{i}/{c_{d}}_{p}$), and the action space is bounded between −1 and 1 through the following transformation,

(7) $${\hat{q}}_{c}=\left\{\begin{array}{ll} \frac{q_{c}-Q_{c}}{Q_{\text{cmax}}-Q_{c}} & \text{if}\;Q_{c}\leq q_{c}\leq Q_{\text{cmax}}\\ \frac{q_{c}-Q_{c}}{Q_{c}-Q_{\text{cmin}}} & \text{if}\;Q_{\text{cmin}}\leq q_{c}\leq Q_{c} \end{array}\right.$$

(8) $$\begin{array}{c} {\hat{q}}_{d,i}=\left\{\begin{array}{ll} \frac{q_{d,i}-Q_{d,i}}{Q_{\text{dmax},i}-Q_{d,i}} & \text{if}\;Q_{d,i}\leq q_{d,i}\leq Q_{\text{dmax},i}\\ \frac{q_{d,i}-Q_{d,i}}{Q_{d,i}-Q_{\text{dmin},i}} & \text{if}\;Q_{\text{dmin},i}\leq q_{d,i}\leq Q_{d,i} \end{array}\right. \end{array}$$

Figure 2b shows the normalized discrete state‐action values corresponding to Equation (1) in the non‐dimensional ${\hat{C_{d}}}_{i},{\hat{q}}_{d,i}$ space evaluated at optimal values along the ${\hat{q}}_{c}$ axis, that is, $\underset{q_{c}}{\text{argmax}}\;R\left({\hat{C_{d}}}_{i},q_{c},q_{d,i}\right)/\underset{q}{\text{argmax}}\;R\left({\hat{C_{d}}}_{i},q_{c},q_{d,i}\right)$. The hashed regions show regions that are off limits due to the imposed constraints. The deterministic policy ($\pi_{d}$) at a given state returns actions with the largest state‐action value, that is, $\pi_{d}\left({\hat{C_{d}}}_{i}\right)=\underset{q}{\text{argmax}}\;R\left({\hat{C_{d}}}_{i},q_{c},q_{d,i}\right)$. This function is shown by the solid line in Figure 2b. In practice, to avoid computing the entire state‐action value matrix, the optimal actions ($q_{c},q_{d,i}$) given by the deterministic policy are recovered via sequential quadratic programming using publicly available libraries.^33^, ^34^

The deterministic control algorithm accelerates drug delivery to the patient, as demonstrated by the dramatic shift in drug delivery profiles from blue curves (w/o control) to the corresponding green curves (Figure 2c). Analytical delivery profiles expected from the deterministic control algorithm agree well with those obtained from experiments and fully resolved numerical simulations, demonstrating the accuracy and robustness of the developed framework. SMART is increasingly effective at lower Sh numbers. As implied in Equation (1) and demonstrated in Figure 2d, the enhancement in drug delivery depends both on parameter $W_{t}$ and the ratio of maximum to steady state infusion drug ($Q_{\text{dmax}}/Q_{d}$) and carrier ($Q_{\text{cmax}}/Q_{c}$) infusion rates. Note even though $Q_{\text{cmax}}/Q_{c}$ can be set independent of $Q_{\text{dmax}}/Q_{d}$, for simplicity, unless otherwise specified we have kept $Q_{\text{cmax}}/Q_{c}=Q_{\text{dmax}}/Q_{d}$. The degree of control depends linearly on $W_{t}$, with no control at $W_{t}=0$ and maximum control at $W_{t}=1$. $Q_{\text{dmax}}/Q_{d}$ determines the maximum enhancement possible. For instance, at $W_{t}=1$ and $Q_{\text{cmax}}/Q_{d}=5$ the time to attain 90% of steady state drug concentration (t90) is 10 times smaller compared to the case without SMART control. These analytical expectations are corroborated by experimental and fully resolved numerical data, as shown in Figure 2e for the case of $Q_{\text{cmax}}/Q_{d}=3$.

The improvement in drug delivery time comes at the price of delivering excess fluid to the patient (Figure 2f). However, since the control algorithms are able to achieve steady state delivery rates by operating for a relatively short duration, the amount of excess fluid delivered is small. For instance, at $W_{t}=1$ and $Q_{\text{cmax}}/Q_{c}=5$, a 10× improvement in drug delivery is achieved by a cumulative excess fluid volume of only about 0.75 mL, which makes this an extremely attractive technology for accelerating drug infusions in both adult and pediatric patients. This framework is scalable to multiple drugs infused in any arbitrary combination of ports (Figure S10).

### 2.5 Deterministically guardrailed reinforcement learning for rapid drug delivery

**FIGURE 3:**
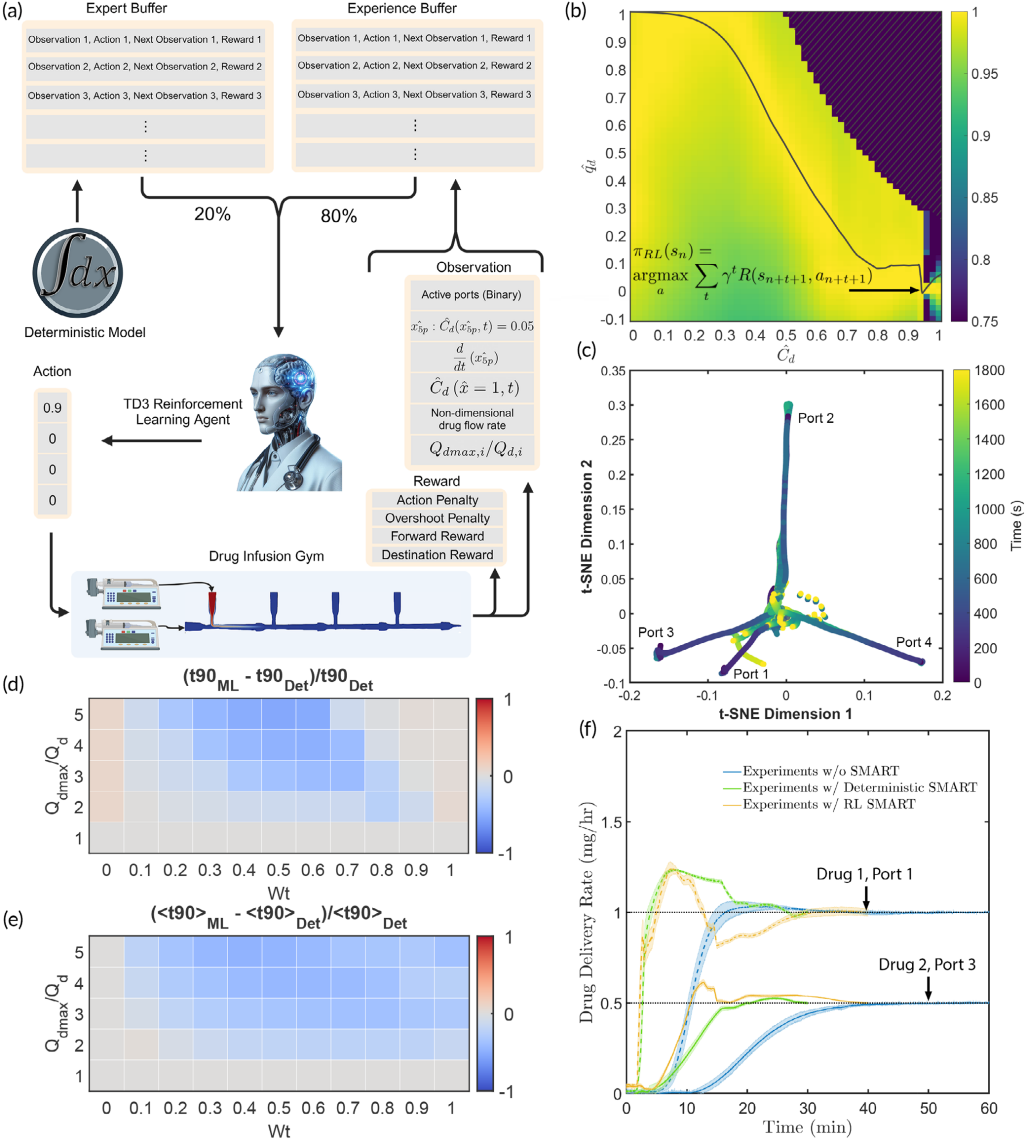
Reinforcement learning (RL) for infusion pump‐based drug delivery. (a) A scalable reinforcement learning architecture for end‐to‐end control of drug infusions utilizing a novel dual buffer strategy—one persistent buffer with expert trajectories obtained from the deterministic model and a first‐in‐first‐out experience buffer. The developed ‘Drug Infusion Gym’ follows the OpenAI gym standard. TD3 agent graphic generated using DALLE. (b) A heat map showing the state‐action‐values learned by RL agent in the $\hat{q_{d}}-\hat{C_{d}}$ plane for single drug delivery ($Q_{\text{dmax}}/Q_{d}=3$). The solid line shows the learned policy $\pi_{\mathrm{RL}}$. (c) The learned policies generate distinct action sequences for different infusion conditions, as confirmed from a t‐SNE embedding of action sequences executed by a trained agent for a single drug injected across different ports ($Q_{\text{dmax}}/Q_{d}=3$). (d) The normalized time difference between the reinforcement learning agent ($t90_{\mathrm{ML}}$) and the deterministic framework ($t90_{\mathrm{ML}}$) in attaining 90% of set concentration as a function of maximum flow rate factor ($Q_{\text{dmax}}/Q_{d}$) and excess fluid weight ($W_{t}$) for a single drug. Blue regions indicate conditions where the reinforcement learning agent outperforms the deterministic framework. (e) The normalized time difference in attaining t90 for all infused drugs when simultaneously administering two drugs. (f) Comparison of multidrug delivery kinetics by SMART running either the deterministic framework or the reinforcement learning framework.

While the deterministic approach gives satisfactory performance for managing drug infusions within manifolds, there are opportunities to further improve drug infusion kinetics with globally optimal approaches, particularly for cases with two or more drugs. Moreover, advances in personalized medicine are increasingly demanding scalable tools for reliably managing drug infusions end‐to‐end to achieve a desired physiological response. Engineering deterministic models to attain these complex objectives is not a scalable approach. Motivated by these needs, we present a scalable reinforcement learning framework for catheter‐mediated drug delivery utilizing a novel dual buffer strategy—one persistent buffer with expert trajectories obtained from the deterministic model and a first‐in‐first‐out experience buffer (Figure 3). This strategy not only helps in balancing forgetting and generalization,^35^ but also enhances the rate of learning and the ultimate performance of the RL agent (Figure S12).

For training the reinforcement learning agent we construct a drug infusion gym following the OpenAI gym standard. The drug infusion gym employs the previously described reduced order drug transport model for physically‐grounded and computationally efficient state evolution of the system in response to an agent's interactions. The agent learns by observing the state of the infusion gym and choosing action that maximize the cumulative rewards. The agent's observation space comprise six state variables, namely a binary representation of the ports with drugs, the non‐dimensional position within the manifold where the drug concentration is at 5% of the set value ($\hat{x_{5p}}$), the velocity of $\hat{x_{5p}}$, the non‐dimensional drug concentration at the catheter tip (${\hat{C}}_{d}\left(1,t\right)$), the non‐dimensional drug flow rate and the ratio $Q_{\text{dmax},i}/Q_{d,i}$. In response to an action, the agent receives a reward comprised of four parts, namely, a penalty for large changes in action, a penalty for overshooting the drug delivery rate above a threshold, a reward for moving the drug closer to the patient and a reward for reaching the desired delivery rate (Figures 3a and S11). In the present implementation, we employ a Twin Delayed Deep Deterministic Policy Gradient (TD3) network as the reinforcement learning agent.^36^ The TD3 agent samples the dual buffer with a 20:80 (expert: experience) split as it learns a policy $\pi_{\mathrm{RL}}$ that maximizes the Bellman equation from a given state $s_{n}$,

(9) $$\begin{array}{c} \pi_{\mathrm{RL}}\left(s_{n}\right)=\underset{a}{\text{argmax}}\;\sum_{t}\gamma^{t}r\left(s_{n+t+1},a_{n+t+1}\right) \end{array}$$

Here γ is the discount factor, t is the time relative to the given state $s_{i}$ and a is the agent's action that specifies the non‐dimensional drug flow rate (Equation 8). The continuous action space is bounded between $\left[1,-1\right]$, with 1, 0, and −1 respectively corresponding to the maximum, set and minimum allowed drug flow rates.

The constructed drug infusion gym allows a TD3 agent to rapidly learn effective policies that respect desired constraints. This can be seen in Figure 3b, where we visualize a part of the state‐action‐value space for a single drug infusion in the $\hat{q_{d}}$–$\hat{C_{d}}$ plane. The hatched area corresponds to regions that violate the constraints specified by Equations ((2), (3), (4), (5), (6)). The solid line shows the action selected by the learned policy $\pi_{\mathrm{RL}}\left(s_{n}\right)$. To ensure safety redundancy, during deployment, the actions of the model are guard railed through a simple deterministic wrapper that enforces the constraints (Figure S11). The learned policies generate distinct action sequences for different infusion conditions. This is evident in Figure 3c, where action sequences generated for a single active drug injected in different ports start off in distinct locations within a t‐SNE embedding, before converging to a common point at long times when the drug delivery has reached the set value (converging to an action of 0 in all ports).

The trained TD3 agents yield comparable performance with the deterministic model for single active drug injection (Figure S13). However, the TD3 agents are dramatically superior to the deterministic model for the more complex case of multidrug delivery, as demonstrated in Figure 3e,f for the case of two drugs. This is a result of the trained TD3 agent's ability to execute optimal decisions (Figure S14) and drive drug infusion trajectories that globally minimize the total drug delivery delay (Figure 3f).

### 2.6 SMART integrated stand‐alone pumps improve drug infusion kinetics and accuracy while reducing clinical workloads

**FIGURE 4:**
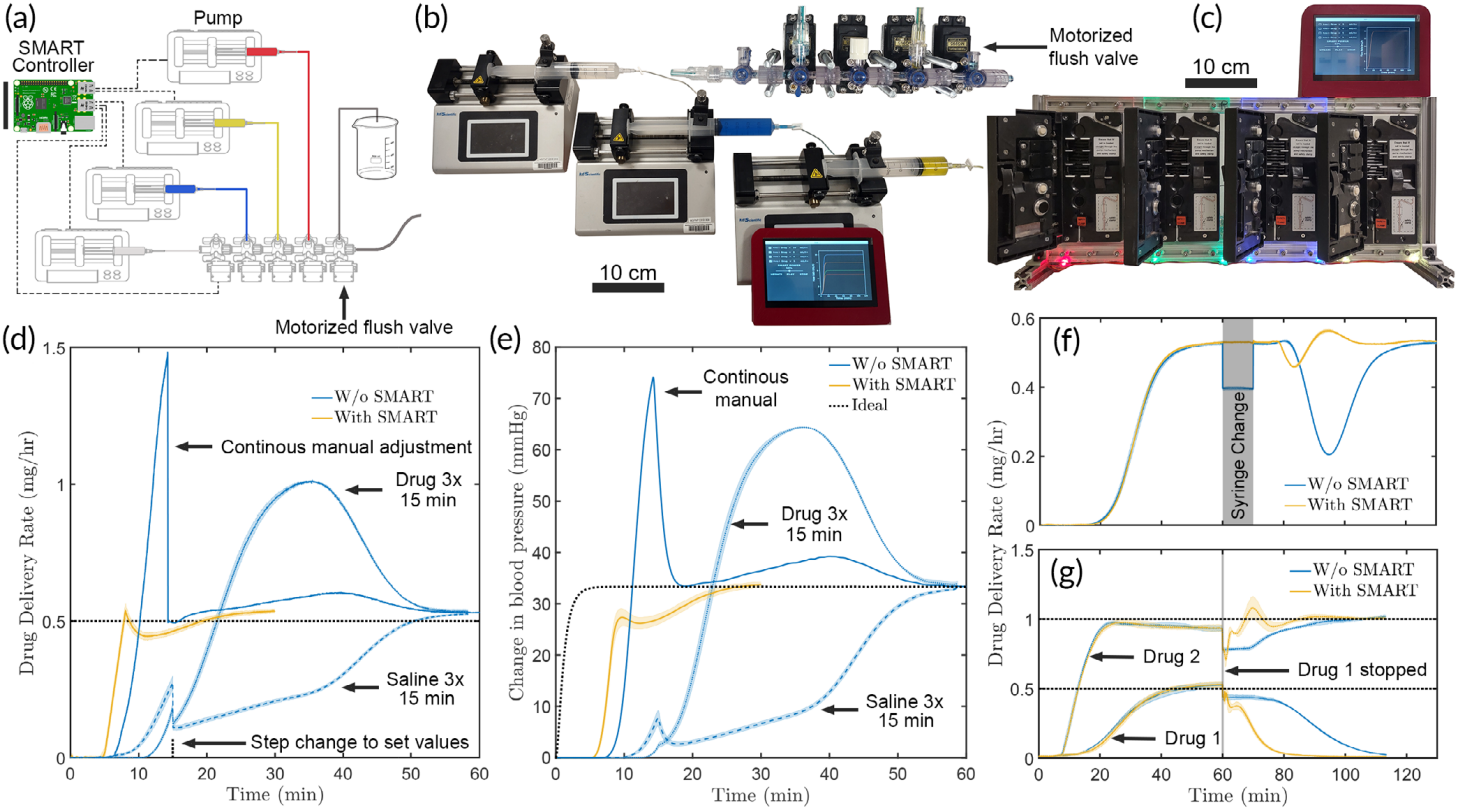
SMART integrated standalone infusion pumps improve drug delivery while reducing clinical workloads and dosing errors. (a) Schematic showing the architecture of SMART integrated infusion pumps delivering drugs through a stopcock assembly with a motorized flush valve. All the infusion pumps and the motorized flush valves are interfaced with a single board computer running SMART. (b) Physical realization of SMART integrated standalone syringe pumps with an intuitive graphical user interface (GUI). (c) Physical realization of SMART integrated standalone peristaltic pumps with an intuitive GUI. (d) Bench top recreation of clinical practices shows that SMART mediated drug delivery (orange curve) is dramatically superior in delivering drugs accurately and rapidly as compared to existing methods of manually managing infusions (blue curves). (e) Physiological response for drug infusion kinetics reported in the previous subfigure obtained via single compartment pharmacokinetic modeling of a fast‐acting vasopressor (parameters selected for phenylephrine). (f) SMART can also mitigate fluctuations in drug delivery kinetics associated with drug container (syringe) changes. g. SMART can also significantly improve the drug cessation kinetics in multi‐drug infusions, with significantly faster cessation of the intended drug (Drug 1) with minimal fluctuation to the other drug (Drug 2).

The computational and memory efficient SMART framework can be deployed in low footprint single‐board computers (SBC) such as the Raspberry Pi (Figure S15). Syringe pumps and any associated accessories such as motorized flush valves (Figure S16) can be interfaced with SBCs running SMART via serial communication ports for synchronized control (Figure 4a). These low footprint SBCs can be integrated into clinical infusion pumps and interfaced with an intuitive graphical user interface (GUI) for realizing clinically compatible standalone SMART infusion pumps. Leveraging the two common infusion technologies used in the clinic, we realized two versions of standalone SMART infusion pumps—one employing syringe pumps (Figure 4b) and the other employing a peristaltic pump (Figure 4c). The infusion mechanism has a minimal impact on the performance of SMART (Figure S17), making this a broadly applicable clinical technology for controlling syringe pumps, peristaltic pumps, or a combination of the two.

SMART dramatically improves drug infusion kinetics and accuracy as compared to the current clinical practice (Figure 4d). Expediting the delivery of drugs in the clinic currently relies on one of the two broad manual approaches: continuously making ad‐hoc adjustments to the pump flow rates or commencing the drug infusion at a high flow rate before initiating a step change to the set flow rates after an arbitrary period of time (Figure S9). As shown in Figure 4d, a bench top recreation of these manual methods reveals their subpar infusion kinetics (t90 as large as 40 min) and accuracy (overshoots as large as 200%) as compared to SMART (t90≈7.5min and negligible overshoots). In addition, the state‐of‐the art manual control methods require extended interventions by clinicians, significantly increasing their cognitive workload. SMART on the other hand requires no manual interventions. The nature of the drug infusion profiles have a direct bearing on the nature of the drug's physiological response. As expected, pharmacokinetic modeling confirms that fast‐acting tightly titrated drugs have a physiological response that mirrors drug infusion kinetics (Figure 4e), highlighting the vital importance of SMART in improving critical care.

SMART can also be interfaced with motorized flush valves to enhance important procedures accompanying multi‐drug infusions such as syringe changes (Figure 4f) and drug cessations (Figure 4g). Syringe changes or exchanges are routinely performed by clinicians to replace depleted drug syringes. This procedure transiently interrupts the infusion of the depleted drug, leading to unwanted instantaneous and delayed fluctuations in drug delivery rates that can adversely affect the physiological parameters of the patient (Figure 4f). SMART can virtually eliminate these fluctuation by proportionally altering the flow rate of the other drugs in the system. Utilizing a similar procedure, SMART can also rapidly cease the infusion of a selected drug with minimal fluctuations in the delivery of the remaining drugs (Figure 4g). In this case, immediately after stopping the infusion of the desired drug, SMART flushes the infusion manifolds so as to reset the concentration to those that should exist for the remaining drugs. This approach reduces $t_{10}$, the time to attain 10% of the existing drug delivery rate, by over 20 min ($t_{10,\mathrm{No}\;\text{Control}}-t_{10,\text{SMART}}\approx 20\;\min$), while simultaneously improving the delivery kinetics of the remaining drugs.

## 3 DISCUSSION

In anesthesia and critical care settings, multiple life‐critical medications formulated in concentrated solutions are commonly administered by medical infusion pumps through manifolds comprising stopcocks and millimeter‐gauge catheters. These critical multidrug infusions currently require careful manual management by clinicians, including starting, stopping, or dose‐adjusting infusions in short time frames while constraining the total fluid delivered to the patient. Experience‐based manual management of infusion pumps is difficult due to the increasingly complex landscape of multidrug infusions brought about by advancements in anesthesia and critical care, such as organ transplantations and neonatal procedures.^5^, ^6^, ^7^, ^8^ The high‐stakes infusion management demands are also complicated by the obscured and coupled transport of drugs within infusion manifolds, which can introduce unpredictable delivery delays and mismatches between clinically intended and actual drug delivery profiles.^37^ These problems, together with the currently strained health care systems with low clinician–patient ratios, demand innovations that can enhance medication delivery accuracy while reducing clinical cognitive workloads. To address this pressing need, we introduce the SMART framework, a drug transport physics‐informed approach that improves multidrug infusions through active synchronized control of infusion pumps.

To adequately deploy SMART, we constructed the Shafer number $\mathrm{Sh}=\tau_{a}Q_{\mathrm{tot}}/V_{\text{dead}}$—a novel non‐dimensional number that quantifies the relative magnitude of a drug's therapeutic action timescale after entering the body ($\tau_{a}$) to the drug's transport timescale within infusion manifolds ($\tau_{p}=V_{\text{dead}}/Q_{\mathrm{tot}}$). When Sh<1, the drug's transport time throttles the effective time for the drug to display its therapeutic effect, leading to delivery delays and confounding the accurate clinical management of multidrug infusions. SMART overcomes these problems by providing automated end‐to‐end management of multidrug infusions through real‐time tracking of drug transport within infusion manifolds, and using this information for synchronized control of infusion pumps to attain desired drug delivery endpoints. SMART obtains spatio‐temporally resolved drug concentration within infusion manifolds in real‐time by leveraging the Gill‐Sankarasubramanian approximation to the advection–diffusion equation with the infusion manifolds approximated through a network model with matched outlet–inlet boundary conditions. This information is leveraged by SMART to provide real‐time optimization of multidrug infusions utilizing an ensemble of deterministic and deep RL‐based decision networks. Notably, during training, this reinforcement learning framework employs a novel dual buffer strategy that integrates deterministic network predicted trajectories with RL agent experience trajectories, enhancing both training speed and performance. For training the RL agent employed by SMART, we also constructed a novel ‘drug infusion gym’ that is compatible with the OpenAI gym standard, promising easy integration within existing deep reinforcement learning workflows.

The computationally efficient SMART framework deployed in standalone infusion pumps enabled real‐time visualization of drug transport within infusion manifolds and significantly improved the drug delivery kinetics while decreasing dosing errors and clinical workloads. In simulated bench top critical care scenarios, the SMART framework outperformed state‐of‐the‐art manual control protocols for reducing delivery delays while eliminating associated dosing errors. The ability to visualize and autonomously manage complex multi‐drug infusions end‐to‐end will not only have important bearings for improving anesthesia and critical care management but is also a key enabling technology for upcoming clinical innovations such as whole organ cryopreservation^38^ and cellular recovery after prolonged warm ischemia.^39^ SMART will also be indispensable for patient care in situations with no or minimal clinical personnel such as in remote, disaster, and conflict zones.^40^, ^41^, ^42^

To safely translate SMART into the clinic, future work should address several outstanding questions. First, preclinical investigations with a broad range of simple and complex (emulsion‐based) drugs are necessary to establish the validity limits of the developed SMART framework. While the developed reduced‐order hydrodynamic model will yield accurate results for simple small‐molecule drugs, additional modifications will be necessary for modeling the flow of less‐common non‐Newtonian drug formulations. Second, comprehensive testing against accidental and adversarial attacks is necessary to establish the reliable operation of reinforcement learning agents within SMART. Even though SMART has minimal entry points for adversarial attacks and is deterministically guardrailed to avoid extreme outcomes, additional testing will be useful in establishing reliability metrics such as the frequency of triggering guardrails and recovery after triggering guardrails.

Beyond clinical settings, the ability for accurate automated end‐to‐end management of multi‐liquid delivery through complex manifolds can address challenges associated with industrial process control, such as improving consistency, reducing waste, and enhancing safety.^43^, ^44^ This capability will also broadly impact continuous flow synthesis applications, including the formulation of nanoparticles, liposomes, biologics, and chemotherapeutics.^45^, ^46^, ^47^ End‐to‐end management of multi‐fluid delivery is also a critical backbone for automated artificial engineering‐driven chemical and material discovery laboratories.^48^, ^49^ Overall, SMART promises end‐to‐end automation of multi‐fluid handling and heralds improved accuracy, efficiency, and safety for applications involving multi‐drug delivery, process control, manufacturing, and beyond.

## 4 METHODS

### 4.1 Model drugs and pumps

Two food‐grade dyes mixed with saline were used as model drugs in our experiments. The food grades were respectively Erioglaucine disodium salt (Sigma Aldrich, 861146‐25G) and Tartrazine (Sigma Aldrich, T0388‐100G).

We used LEGATO® 110 manufactured by KD Scientific for our syringe pump experiments (Figure 4b). The four‐channel peristaltic pumps (Figure 4c) were constructed utilizing linear peristaltic units extracted from Baxter 6200 pumps.

### 4.2 In vitro bench top spectrophotometric setup

The bench top spectrophotometric setup used for measuring model drug concentrations at the catheter tip consists of two high intensity light sources capable of emitting light at wavelengths of 630 nm $\left(I_{630}\right)$ and 430 nm $\left(I_{430}\right)$ (Thorlabs, New Jersey, USA—model M625l4 and M430l4 respectively). Both LEDs are integrated into our system using a Thorlabs C4W 30 mm Cage Cube, which includes a dichroic mirror (Thorlabs DMLP505R) and a specialized lens (Thorlabs LA4647). This assembly is able to combine and converge the light beams from both LEDs and couple them to the inlet optical fiber cable that is connected to a Z‐flow cell (Ocean Insight, Florida, USA, model FIA‐ZSMA‐SS). The LEDs are powered and controlled by a Thorlabs LEDD1B T‐Cube LED Driver, allowing for precise modulation of light intensity. The emitted light enters the flow cell through an optical fiber, passes through the fluid in the optical path before being transmitted to the spectrometer via another optical fiber. The employed Thorlabs CCS100 Compact Spectrometer was used to record intensities in the wavelength range spanning 350–700 nm with an integration time of 1000μs. The recorded spectrophotometric data was processed as detailed in Figure S4 to recover the temporally resolved drug concentration at the catheter tip (make and model). A digital scale (Ainsworth M‐220, USA) was used to measure the total dispensed fluid mass as function of time. This data was further processed to obtain the total infused flow rates.

### 4.3 In silico OpenFOAM fully resolved numerical simulations

To extract the fluid volumes for simulation, the infusion manifolds were microCT scanned (X‐Tek—HMXST225) and processed in ORS DragonFly (Comet Technologies, Montreal, Canada). The extracted 3D volume was saved as an STL file and appropriately meshed using snappyHexMesh (OpenFOAM) to obtain mesh and time step independent results (Figure S5). To perform multidrug infusion simulations, we modified the OpenFOAM IcoFoam solver to include a generalized advection–diffusion equation for solving the transport of a passive scalar with adaptive time stepping. The simulations were parallelized by decomposing the meshed geometry using decomposePar (OpenFOAM) and run across 30 compute cores.

### 4.4 OpenAI standardized infusion gym environment

The infusion gym constructed to train the SMART reinforcement learning agent follows the OpenAI gym standard. The infusion gym consists of a 24 × 1 observation space and a 4 × 1 action space (Figure 3). The standard methods include step(action): for evolving the environment through one time step, reset(): for resetting the environment to the initial state, and render(): for visualizing the actions of the agent. Additional functionalities include routines to evoke the deterministic expert trainer and run a trained agent.

### 4.5 Statistical analysis

Data in all plots are expressed as mean ± *SD* of three or more independent measurements or trials unless otherwise mentioned.

## AUTHOR CONTRIBUTIONS

**V. Chandran Suja:** Conceptualization; methodology; software; data curation; investigation; validation; formal analysis; visualization; writing – original draft; writing – review and editing. **A. L. H. S. Detry:** Methodology; data curation; investigation; validation; formal analysis; visualization; writing – original draft; writing – review and editing. **N. M. Sims:** Conceptualization; supervision; writing – review and editing. **D. E. Arney:** Conceptualization; methodology; supervision; project administration; resources; writing – review and editing; funding acquisition. **S. Mitragotri:** Conceptualization; funding acquisition; project administration; resources; writing – review and editing; supervision. **R. A. Peterfreund:** Conceptualization; supervision; funding acquisition; project administration; resources; writing – review and editing.

## CONFLICT OF INTEREST STATEMENT

V.C.S., A.L.H.S.D., N.M.S., R.A.P., S.M., and D.E.A. are inventors on a patent application related to the technology described in this manuscript (owned and managed by Mass General Hospital & Harvard University).

## Supporting information


**APPENDIX S1.** Supporting information.

**TABLE S1.** Therapeutic action timescale of common critical drugs. Carrier flow rate: adult—10 mL/h and pediatric—1.5 mL/h, dead volume: adult—1.2 mL and pediatric—0.7. Patient weight: adult—70 kg and pediatric—7 kg. For calculating the Shafer number, the lower bound of the delivery was used.

## Data Availability

The data that supports the findings of this study are available in the supplementary material of this article.
