# Supplementary material for: Transport physics‐informed reinforcement learning agents deployed in standalone infusion pumps for managing multidrug delivery in critical care
Source: Bioeng Transl Med. 2025 Mar 18;10(5):e70013. doi: 10.1002/btm2.70013 (PMC12478332; doi:10.1002/btm2.70013)
Supplement: Supplementary file 1 — APPENDIX S1. Supporting information. [file BTM2-10-e70013-s002.pdf]

Supplementary Information for

Transport physics informed reinforcement learning agents deployed in standalone infusion pumps for managing multidrug delivery in critical care

V. Chandran Suja; A. L. H. S. Detry; N. Sims; D. E. Arney; S. Mitragotri; R. A. Peterfruend

D. E. Arney (darney@mgh.harvard.edu)
S. Mitragotri (mitragotri@seas.harvard.edu)
R. A. Peterfruend (rpeterfruend@mgh.harvard.edu)

This PDF file includes:

Supplementary text
Figs. S1 to S17
Tables S1 to S2
References for SI reference citations

Supporting Information Text

Fluid Management Card

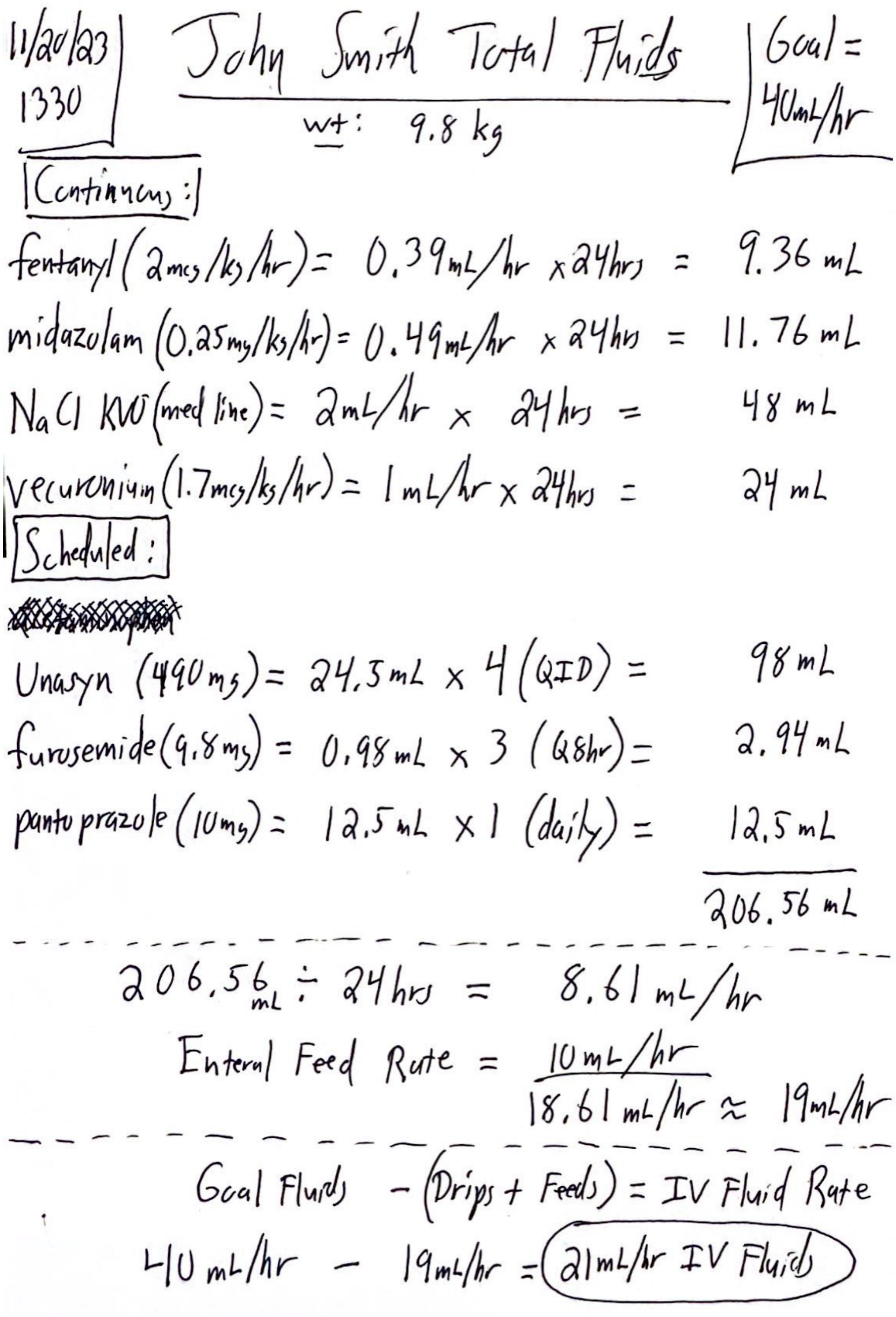
11/20/23
1330

John Smith Total Fluids

wt: 9.8 kg

Goal = 40mL/hr

Continuous:

fentanyl (2mcg/kg/hr) = 0.39mL/hr x 24hrs = 9.36 mL

midazolam (0.25mg/kg/hr) = 0.49mL/hr x 24hrs = 11.76 mL

NaCl KVO (med line) = 2mL/hr x 24 hrs = 48 mL

vecuronium (1.7mcg/kg/hr) = 1 mL/hr x 24hrs = 24 mL

Scheduled:

[illegible]

Unasyn (490mg) = 24.5mL x 4 (QID) = 98mL

furosemide (9.8mg) = 0.98mL x 3 (Q8hr) = 2.94mL

pantoprazole (10mg) = 12.5mL x 1 (daily) = 12.5mL

206.56 mL

206.56 mL ÷ 24hrs = 8.61 mL/hr

Enteral Feed Rate = 10mL/hr

18.61 mL/hr ≈ 19mL/hr

Goal Fluids − (Drips + Feeds) = IV Fluid Rate

40 mL/hr − 19mL/hr = 21mL/hr IV Fluids

Fig. S1. A typical fluid management card used in the clinic to manage infusions. The numbers correspond to those seen in pediatric intensive care units

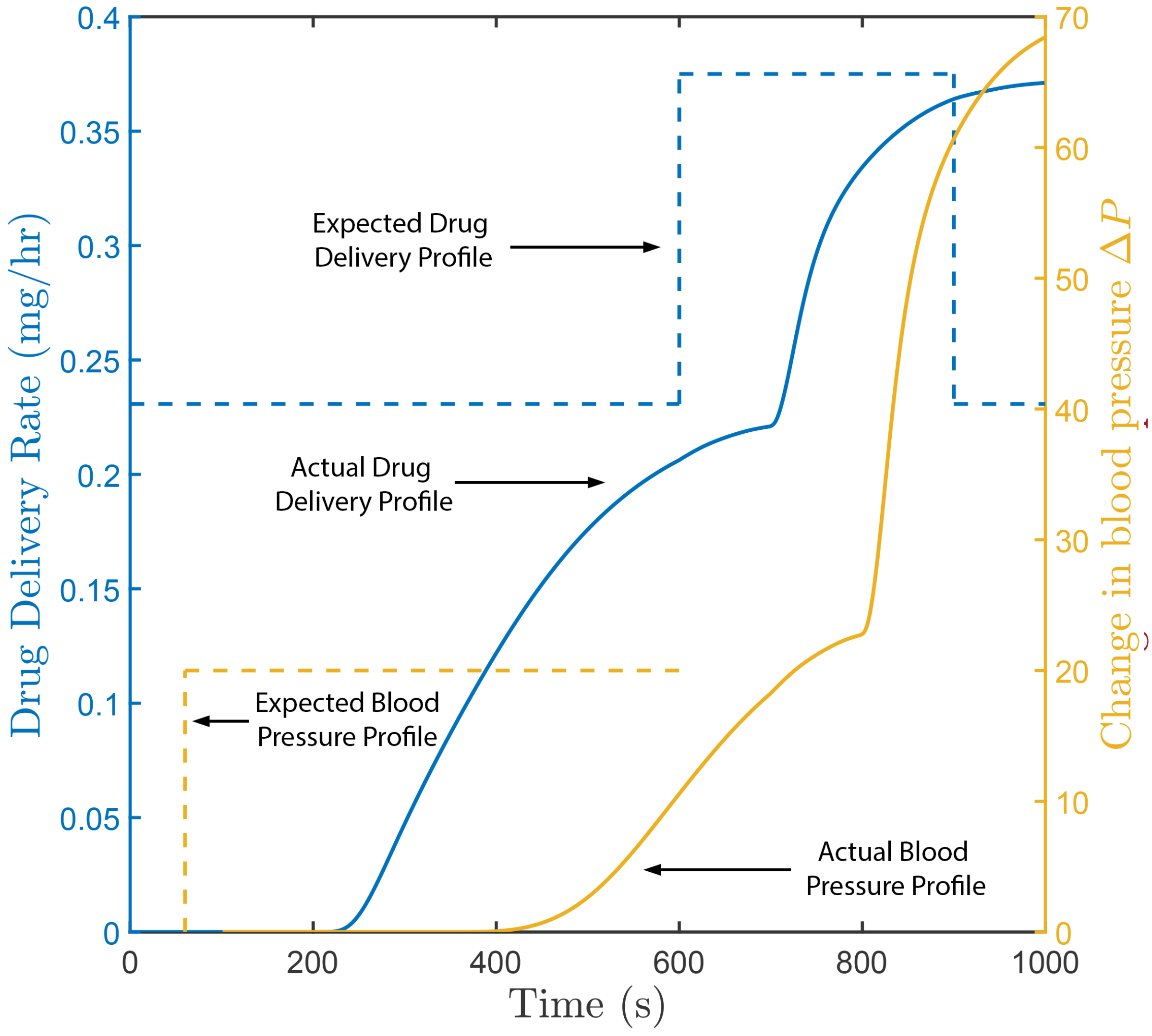

Fig. S2. Drug delivery delays can confound the management of critical physiological parameters - a numerical recreation of a common clinical drug delivery delay problem.

This numerical simulation of a common clinical scenario assumes a fictitious clinically relevant set drug flow rate and corresponding expected change in blood pressure. The set flow rate was imposed on a reduced order numerical simulation (as described in the main text) with the drug injected in port 3 to obtain the actual drug delivery profile. The mathematical correlation below was then used to link blood pressure to the drug concentration(1, 2):

$$\Delta P = \frac{100}{1 + (0.3/c_d)^{-4}} \tag{1}$$

Time scales and the Shafer number

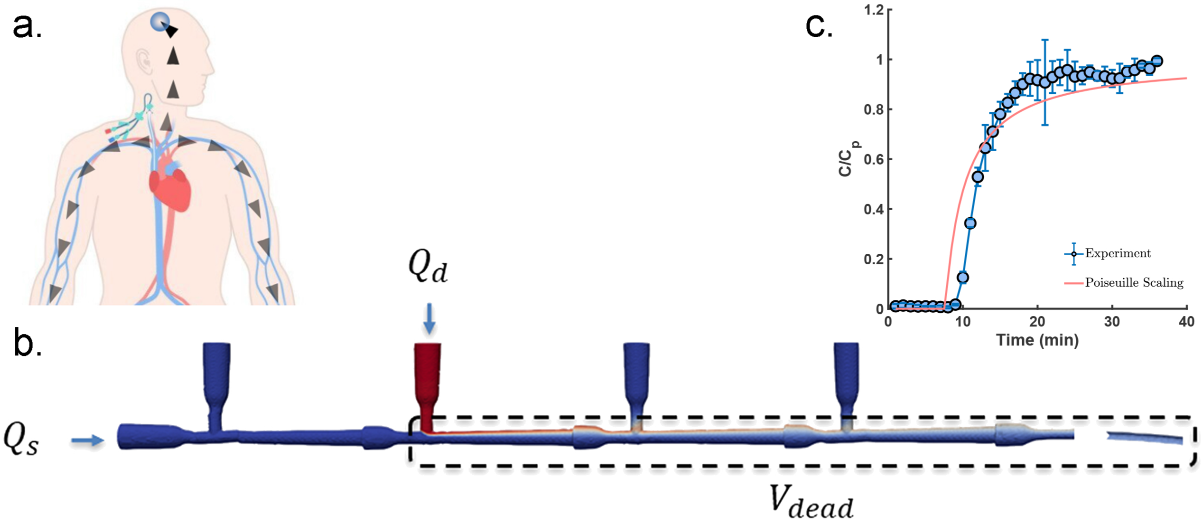

Fig. S3. The importance of synchornized-pump control for mitigating drug delivery delays depends on the relative magnitude of the drug absorption timescale (τ_a) and the Poiseuille timescale (τ_p). (a.) Schematic showing the PK of an administered drug that dictates (τ_a). (b.) The variables that dictate τ_p during catheter mediated delivery of drugs. (c.) Concentration evolution as predicted by the Poiseuille timescale.

There are two important time scales that dictate how quickly a drug acts. A drug administered as a bolus takes time depending on its PK characteristics to partition in to the tissues of interest, and act on appropriate cellular targets to elicit the appropriate biochemical response (Fig.S3a). Let us call this drug action timescale as τ_a.

Another timescale enters the problem purely due to the advective transport of the drug within the catheter (Fig.S3b). For a catheter geometry with a dead volume V_{dead}, and total drug flow rate $Q_{tot} = Q_s + Q_d$, the timescale for the drug to reach the catheter tip can be shown from Poiseulle flow kinetics to scale as,

$$\tau_p = \frac{V_{dead}}{Q_{tot}} \qquad [2]$$

and the concentration to scale as,

$$\hat{C} = c/c_p = \begin{cases} 0 & \text{if } t < \tau_p \\ 1 - \tau_p/(2t) & \text{if } t \geq \tau_p \end{cases} \qquad [3]$$

Here c_p is the plateau or the steady-state drug concentration at the tip of the catheter. The factor of 2 comes from the fact that max velocity in Poisuelle flow is twice the mean velocity.

Controlling syringe pumps become important when $\tau_a/\tau_p < 1$. There are two scenarios where this can happen in the clinic - (a) for fast acting drugs such as anesthetics and vassopressors, and (b) for infusions at small flow rates such as in neonatal care.

Therapeutic action timescales and deliver information for common drugs

No.	Name	τ_a (min)	Stock conc. (mcg/ml)	Delivery rates
1	Remifentanil	0.5 – 1 (3)	50	$0.05 - 0.2\ mcg/kg/min$
2	Ketamine	0.5 (4)	10000	$2 - 10\ mcg/kg/min$
3	Norepinephrine	5 (5)	128	$0.03 - 0.3\ mcg/kg/min$
4	Dexmedetomidine	10 (6)	4	$0.2 - 1.0\ mcg/kg/hr$
5	Vasopressin	15 (7)	1 (unit)	$0.04 - 1.0\ units/minute$
6	Nitroglycerine	1 (8)	400	$0.15 - 2.86\ mcg/kg/ml$
7	Nicardipine	5 (9)	250	$0.035 - 0.2\ mg/kg/hr$
8	Clevidipine	2 (10)	500	$1 - 21\ mg/hr$
9	Phenylephrine	1 (11)	80	$0.286 - 4.4\ mcg/min$
10	Propofol	0.7 (12)	10000	$100 - 200\ mcg/kg/min$
11	Sufentanil	1.5 (13)	10	$0.1 - 0.4\ mcg/kg/hr$
12	Furosemide	30 (14)	10000	$100\ mcg/kg/hr$
13	Amiodarone	60 (15)	50000	$0.5\ mg/min$
14	Epinephrine	1 (16)	8	$0.0071 - 0.1714\ mcg/kg/ml$

Table S1. Therapeutic action timescale of common critical drugs. Carrier flow rate: Adult - $10\ ml/hr$ **and Pediatric -** $1.5\ ml/hr$**, Dead volume: Adult -** $1.2\ ml$ **and Pediatric -** 0.7**. Patient weight: Adult** $70\ kg$ **and Pediatric** $7\ kg$**. For calculating the Shafer number, the lower bound of the delivery was used.**

The Shafer number computed from data in Tab.S1 is reported in the main text Fig. 1b.

Spectrum and Calibration Curves of Model Drugs

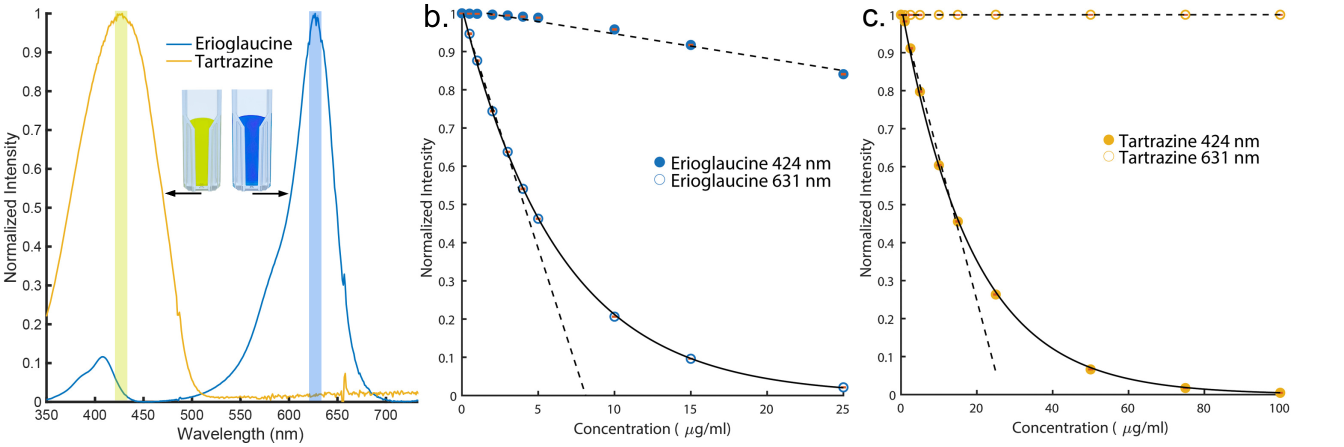

Fig. S4. **a.** Absorption spectrum of Erioglaucine and Tartrazine. Concentration versus normalized transmission intensity calibration curves for Erioglaucine **b.** and Tartrazine **c.** Solid curves correspond to single exponential fits while dashed lines correspond to linear fits. All points were obtained from three different measurements and the error bar from their standard deviation.

We determine the concentrations of Tartrazine (C_{Tar}) and Erioglaucine (C_{Meth}) at the tip of the catheter by measuring their transmissivity at selected wavelengths. An experimental setup composed of a light emitter, a flow cell and a spectrophotometer is suitable for this purpose. Two light sources were used in this study with wavelengths of 630 nm (I_{630}) and 430 nm (I_{430}).

Fig S4b shows the calibration curve for Erioglaucine. The curve is derived from measuring how different concentrations of Erioglaucine dye affect the light intensity at these wavelengths. The solid dots represent measurements at 424 nm, while the open blue circles correspond to 631 nm. As the concentration of Erioglaucine increases, the normalized light transmission intensity decreases due to increased absorbance by the dye. This is consistent with Beer-Lambert law, which states that the absorbance of a substance is directly proportional to its concentration. By fitting these data points, we get equations (Eq.4, Eq.5) that describe how the intensity of light at each wavelength changes with Erioglaucine concentration. Similarly, the calibration curves for Tartrazine are established by measuring the transmitted light intensity as function of concentration at the two wavelengths. Figure S4c presents the calibration data for Tartrazine. Fitting these data points yielded Eq.6 and Eq.7.

$$I_{630M} = \exp(-1.0712 \cdot C_{Meth}) \quad [4]$$
$$I_{430M} = -0.0532 \cdot C_{Meth} + 1 \quad [5]$$
$$I_{430T} = \exp(-0.0691 \cdot C_{Tar}) \quad [6]$$
$$I_{630T} = -0.00001339 \cdot C_{Tar} + 1 \quad [7]$$

To determine the concentrations C_{Tar} and C_{Meth}, one must solve a system of coupled equations based on the experimentally measured resultant intensities (I_{631}) and (I_{424}) at the two respective wavelengths such that,

$$\begin{cases} I_{631} = I_{630T} - I_{630M} \\ I_{424} = I_{430T} - I_{430M} \end{cases} \quad [8]$$

We use MATLAB's **fsolve** function to numerically solve the system of equations, and recover the true concentration C_{Tar}) and (C_{Meth}).

Mesh and Time step independence

To evaluate the dependence of mesh characteristics on the numerical results, four different meshes were created with the number of cells varying an order of magnitude from $50K$ to $550K$. Identical single drug test cases were run on all the meshes with carrier and drug stream flow rates of 10 ml/hr and 3 ml/hr respectively (see Table S2 for more details). The evolution of single drug concentration at the tip of the catheter was measured to evaluate mesh independence (Fig.S5). The negligible difference in concentration profiles between meshes having $\sim 350K$ and $\sim 550K$ elements indicates that numerical mesh is achieved at a size of $\sim 350K$. This mesh was used for all subsequent calculations reported in the manuscript.

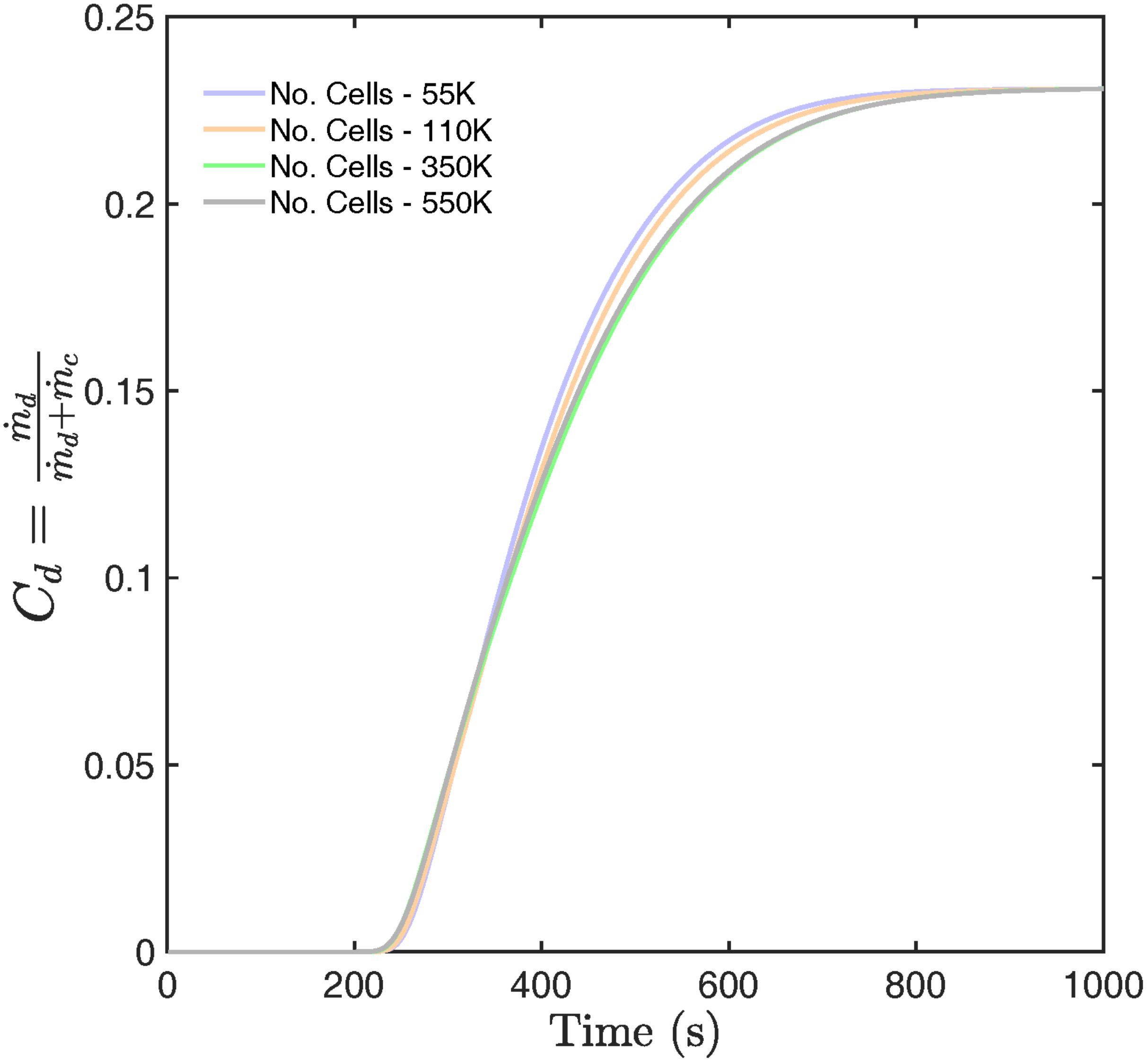

Fig. S5. The evolution of single drug concentration at the tip of the catheter for four different mesh with varying degrees of refinement. The carrier stream was injected at $10; ml/hr$ while the drug was injected at $3; ml/hr$. The results show that mesh Independence is achieved at a size of $\sim 350K$.

No. Cells	Single Core Equivalent Clock Time (s)	$C_d(t = 500s)$	t_{cd90}
55K	17664	0.1905	555
110K	46500	0.1858	570
350K	305494	0.1793	597
550K	n/a	0.1776	595

Table S2. Mesh independence study metrics

Improving accuracy over the Taylor diffusion approximation via a temporally varying diffusion coefficient

Following Gill and Sankarasubramanian (17), we express the effective diffusion coefficient as a function of time as follows,

$$D_{e,i}(\tau_i) = D_i\left(\frac{Pe_i^2}{48} + 1 - 16Pe_i^2\Sigma_{n=1}^{\infty}B_n \exp -\lambda_n^2\tau_i\right) \quad [9]$$

where,

$$B_n = \frac{J_3(\lambda_n)J_2(\lambda_n)}{\lambda_n^5[J_0(\lambda_n)]^2} \quad [10]$$

$$J_1(\lambda_n) = 0 \quad [11]$$

$$\tau_i = \frac{D_i t}{a^2} \quad [12]$$

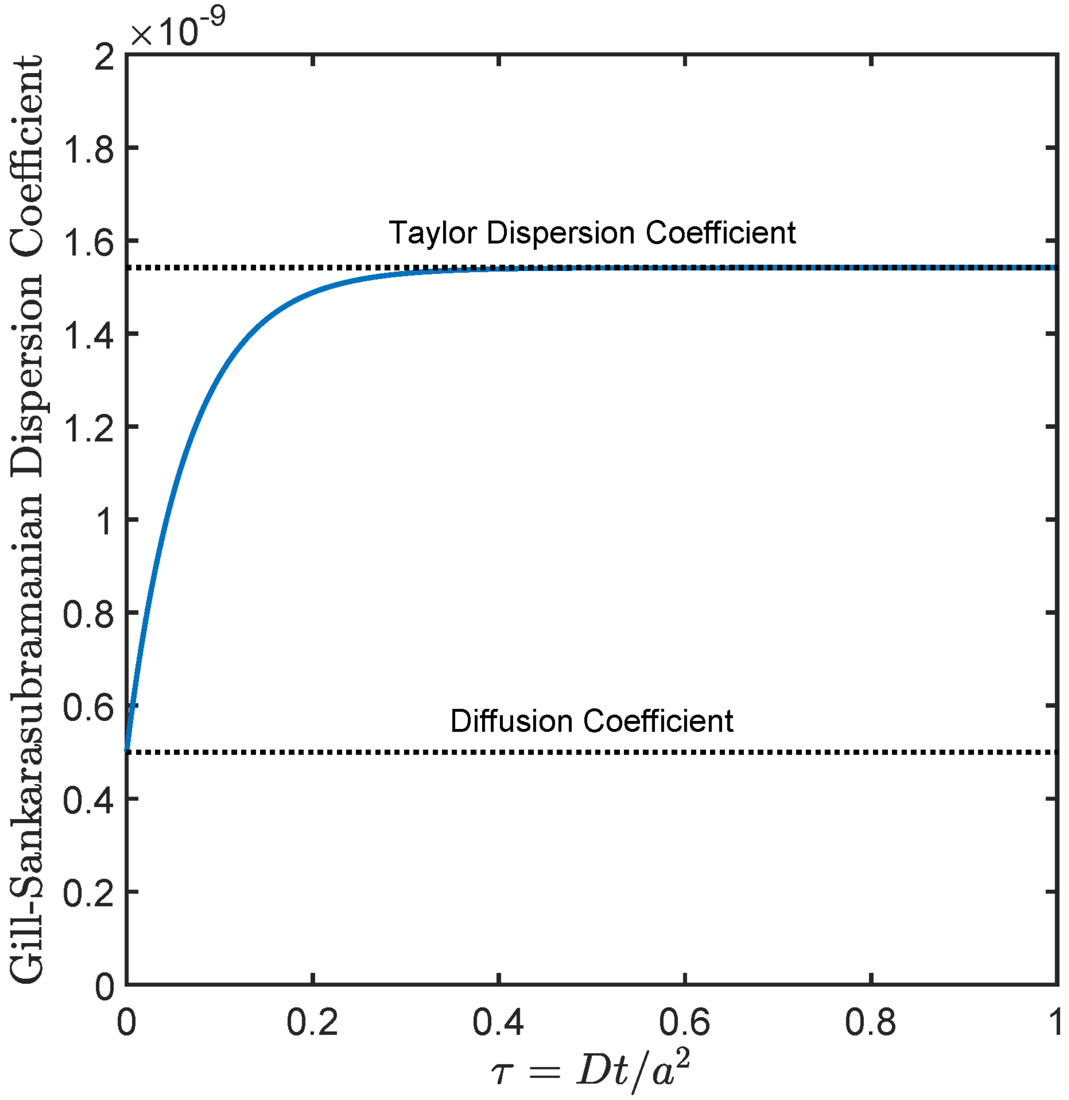

Fig. S6. Temporally varying diffusion coefficient obtained via the Gill-Sankarasubramanian approximation smoothly evolves from the intrinsic diffusion coefficient of the liquid to Taylor dispersion coefficient over time.

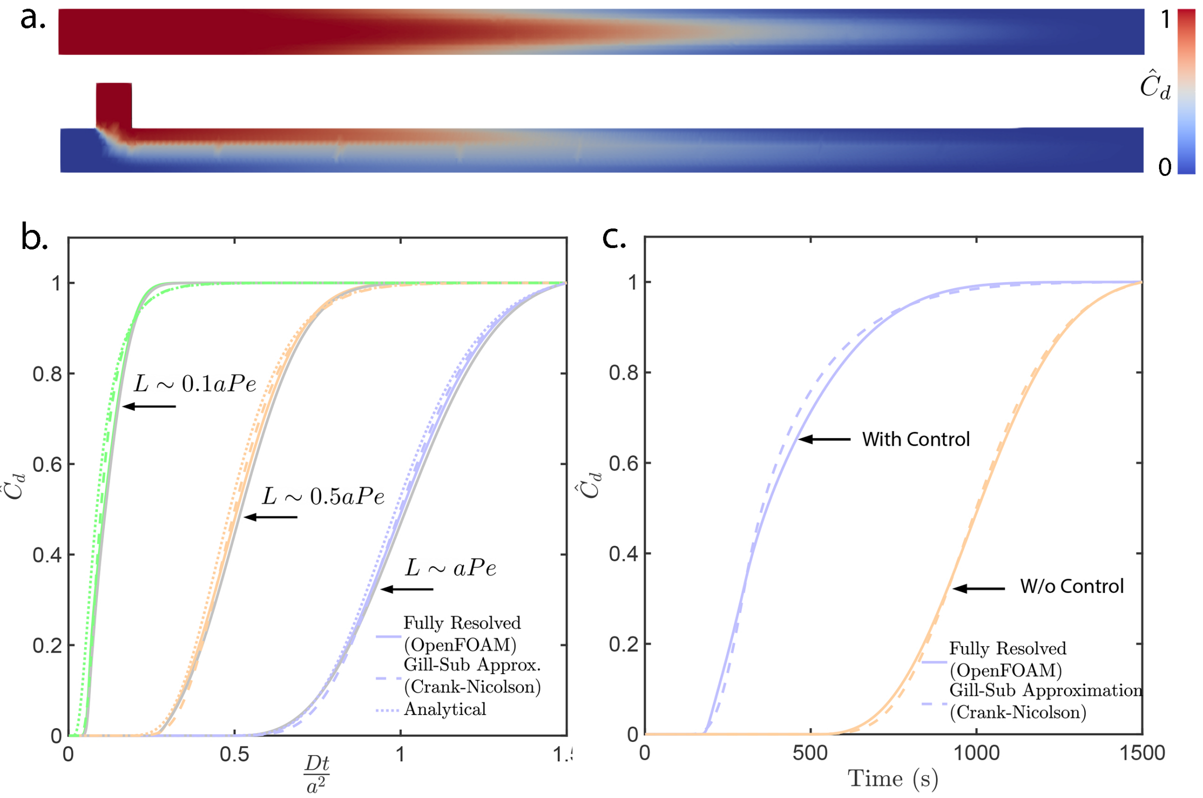

Fig. S7. Validation of developed algorithmic framework on canonical geometries. **a.** Spatio-temporal drug concentration profiles obtained from fully resolved simulations using OpenFOAM. **b.** Normalized drug concentration evolution at different points along the length of the cylindrical geometry for steady convection. Here $\hat{C}_d = cd/c_{d,p}$ is the non-dimensional concentration normalized with the plateau or steady state concentration. **c.** Normalized drug concentration evolution at end of geometry where the drug introduced orthogonal to the carrier stream. Here W/o control has steady convection while With control has unsteady convection.

The advective transport of drugs within catheters is described by the convection-diffusion equation,

$$\frac{dc_{d,i}}{dt} + \mathbf{U}.\nabla c_{d,i} = D_i \nabla^2 c_{d,i} \qquad [13]$$

where $c_{d,i}$ is the concentration and D_i is the diffusion coefficient of the ith drug in the catheter. The velocity field $\mathbf{U}$ satisfies the Navier-Stokes equation,

$$\frac{d\mathbf{U}}{dt} + \mathbf{U}.\nabla \mathbf{U} = -\frac{1}{\rho}\nabla p + \nu \nabla^2 \mathbf{U} \qquad [14]$$

Analytical solutions are tractable for Eq.13 for unidirectional velocity fields. For a Poiseulle flow field in cylindrical co-ordinates $\mathbf{U} = u(r)$, Eq.13 can be expressed in terms of average quantities as,

$$\frac{\partial \bar{c}_{d,i}}{\partial t} + \bar{u}\frac{\partial \bar{c}_{d,i}}{\partial z} = D_i\left(\frac{Pe_i^2}{48} + 1\right)\frac{\partial^2 \bar{c}_{d,i}}{\partial z^2} \qquad [15]$$

where quantities with $\bar{.} = \frac{1}{R}\int_0^R .dr$, and $Pe_i = \bar{u}_i R/D_i$.

$$\bar{c}_{d,i} = \frac{\bar{c}_{d,i,o}}{2} \mathrm{e}^{\frac{\bar{u}z}{D_{e,i}}} \left[\mathrm{erfc}\left(\frac{z + \bar{u}t}{2\sqrt{D_{e,i}t}}\right) + \mathrm{e}^{-\frac{\bar{u}z}{D_{e,i}}}\mathrm{erfc}\left(\frac{z - \bar{u}t}{2\sqrt{D_{e,i}t}}\right)\right] \qquad [16]$$

where $D_{e,i} = D_i\left(\frac{Pe_i^2}{48} + 1\right)$ is the Taylor-Diffusion coefficient (or the effective diffusion coefficient) of the ith drug and $\bar{c}_{d,i,o}$ is the radial-average initial concentration of the ith drug.

Reduced order modeling improves computational time by a factor exceeding 10^4

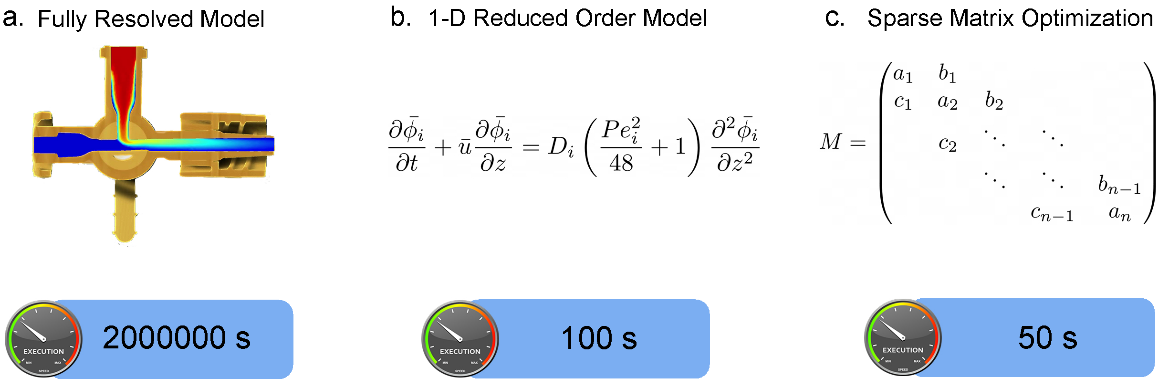

Fig. S8. Computational time required for simulating 1000 seconds of drug infusion for different computational models - **a.** Fully resolved model solved using OpenFOAM, **b.** 1-D reduced order model employing Taylor diffusion approximation solved using MATLAB, and **c.** Computationally optimized reduced order model solved using MATLAB.

Infusion scenarios reported in the manuscript

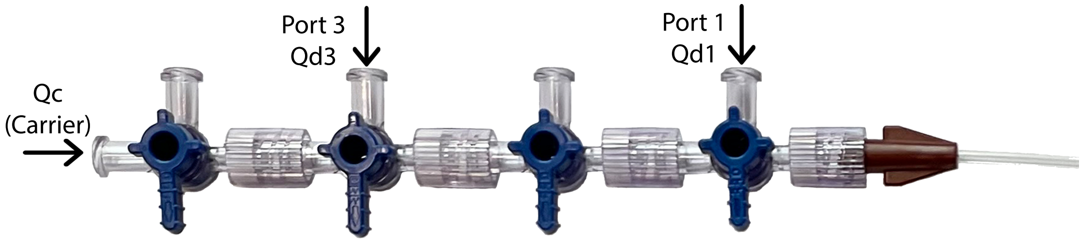

Fig. S9. Drug infusion conditions reported in the manuscript

A. Drug delivery without SMART.

Single drug Adult, Port 3:
$Q_c = 10$ ml/hr
$Q_{d3} = 3$ ml/hr

Single drug Pediatric, Port 1 & 3:
$Q_c = 1.5$ ml/hr
$Q_{d1} = 0.5$ ml/hr

Two drugs Pediatric:
$Q_c = 1.5$ ml/hr
$Q_{d1} = 1.0$ ml/hr
$Q_{d3} = 0.5$ ml/hr

B. Drug delivery with SMART.

Single drug Adult, Port 3:
$Q_c = 10$ ml/hr $\quad Q_{cmax} = 30$ ml/hr
$Q_{d3} = 3$ ml/hr $\quad Q_{d3max} = 10$ ml/hr

Single drug Pediatric, Port 1 & 3:
$Q_c = 1.5$ ml/hr $\quad Q_{cmax} = 4.5$ ml/hr
$Q_{d1} = 0.5$ ml/hr $\quad Q_{d1max} = 1.5$ ml/hr

Two drugs Pediatric:
$Q_c = 1.5$ ml/hr $\quad Q_{cmax} = 4.5$ ml/hr
$Q_{d1} = 1.0$ ml/hr $\quad Q_{d1max} = 3.0$ ml/hr
$Q_{d3} = 0.5$ ml/hr $\quad Q_{d3max} = 1.5$ ml/hr

C. Manual control experiments.

Continuous manual adjustment
$Q_c = 1.5$ ml/hr
$Q_{d3} = 0.5$ ml/hr
$Q_{cmax} = 4.5$ ml/hr
$Q_{d3max} = 1.5$ ml/hr

Drug 3X 15 min
$Q_c = 1.5$ ml/hr
$Q_{d3} = 1.5$ ml/hr
After 15 min:
$Q_c = 1.5$ ml/hr
$Q_{d3} = 0.5$ ml/hr

Saline 3X 15 min
$Q_c = 4.5$ ml/hr
$Q_{d3} = 0.5$ ml/hr
After 15 min:
$Q_c = 1.5$ ml/hr
$Q_{d3} = 0.5$ ml/hr

D. Syringe change experiment without SMART.

First step:
$Q_c = 1.5$ ml/hr
$Q_{d3} = 0.5$ ml/hr

After 60min:
$Q_c = 1.5$ ml/hr
$Q_{d3} = 0$ ml/hr

After 70min:
$Q_c = 1.5$ ml/hr
$Q_{d3} = 0.5$ ml/hr

E. Syringe change experiment with SMART.

First step:
$Q_c = 1.5$ ml/hr
$Q_{d3} = 0.5$ ml/hr

After 60min:
$Q_c = 2$ ml/hr
$Q_{d3} = 0$ ml/hr

After 70min open the flush valve:
$Q_c = 450$ ml/hr
$Q_{d3} = 150$ ml/hr

After 70.5 min(30sec of flushing) close the flush valve:
$Q_c = 1.5$ ml/hr
$Q_{d3} = 0.5$ ml/hr

F. Drug cessation experiments without SMART.

First step:
$Q_c = 1.5$ ml/hr
$Q_{d1} = 1.0$ ml/hr
$Q_{d3} = 0.5$ ml/hr
After 60min:
$Q_c = 1.5$ ml/hr
$Q_{d1} = 1.0$ ml/hr
$Q_{d3} = 0$ ml/hr

G. Drug cessation experiments with SMART.

First step:
$Q_c = 1.5$ ml/hr
$Q_{d1} = 1.0$ ml/hr
$Q_{d3} = 0.5$ ml/hr

After 60 min open the flush valve:
$Q_c = 450$ ml/hr
$Q_{d1} = 450$ ml/hr
$Q_{d3} = 0$ ml/hr

After 60.5 min(30sec of flushing) close the flush valve:
$Q_c = 1.5$ ml/hr
$Q_{d1} = 1.0$ ml/hr
$Q_{d3} = 0$ ml/hr

Deterministic policy for SMART. The deterministic policy simply chooses the action $(q_c, q_{d,i})$ the maximizes the reward function at the current non-dimensional state $(\hat{C}d_i = cd_i/cd_{i,p})$. The reward function is physically connected to the error between the expected and actual drug delivery rates, and is defined as below:

$$R(\hat{C}d_i, q_c, q_{d,i}) = -\exp\left(\left(Q_c + \sum_{n=1}^{i} Q_{d,i}\right) cd_{i,p} - \left(q_c + \sum_{n=1}^{i} q_{d,i}\right) cd_i\right) - (1+\hat{C}d_i)^2(1-W_t)(Q_c-q_c)^2 - (1-\hat{C}d_i)^2 W_t (Q_{cmax}-q_c)^2 \quad [17]$$

In the first term on the RHS, we can factor out $cd_{i,p}$. Since we are only interested in the extrema of each term, we can neglect the constant factor $cd_{i,p}$ yielding the expression,

$$R(\hat{C}d_i, q_c, q_{d,i}) = -\exp\left(\left(Q_c + \sum_{n=1}^{i} Q_{d,i}\right) - \left(q_c + \sum_{n=1}^{i} q_{d,i}\right) \hat{C}d_i\right) - (1+\hat{C}d_i)^2(1-W_t)(Q_c-q_c)^2 - (1-\hat{C}d_i)^2 W_t (Q_{cmax}-q_c)^2 \quad [18]$$

Subject to,

$$0 \leq q_c \leq Q_{cmax} \quad [19]$$

$$0 \leq q_{d,i} \leq Q_{dmax,i} \quad [20]$$

$$\frac{q_{d,i}}{q_{d,i} + q_c} \leq (1+\kappa)\frac{Q_{d,i}}{Q_{d,i} + Q_c} \quad [21]$$

$$\frac{q_{d,i}}{q_{d,i} + q_c} \geq (1-\kappa)\frac{Q_{d,i}}{Q_{d,i} + Q_c} \quad [22]$$

$$\left(q_c + \sum_{n=1}^{i} q_{d,i}\right)\hat{C}d_i \leq \beta\left(Q_c + \sum_{n=1}^{i} Q_{d,i}\right) \quad [23]$$

Here $Q_{d,i}$ and $Q_{dmax,i}$ are the steady state and maximum allowable flow rate of the ith drug, Q_c and Q_{cmax} are the steady state and maximum allowable flow rate of carrier, κ is the maximum permissible percent fluctuation in concentration at the inlet, β multiplicative factor determining the maximum permissible overshoot in drug concentration at the outlet.

For improved stability during nonlinear optimization and for machine learning based algorithm development, we normalize the action variables as below.

$$\hat{q}_c = \begin{cases} \frac{q_c - Q_c}{Q_{cmax} - Q_c} & \text{if } Q_c \leq q_c \leq Q_{cmax} \\ \frac{q_c - Q_c}{Q_c - Q_{cmin}} & \text{if } Q_{cmin} \leq q_c \leq Q_c \end{cases} \quad [24]$$

$$\hat{q}_{d,i} = \begin{cases} \frac{q_{d,i} - Q_{d,i}}{Q_{dmax,i} - Q_{d,i}} & \text{if } Q_{d,i} \leq q_{d,i} \leq Q_{dmax,i} \\ \frac{q_{d,i} - Q_{d,i}}{Q_{d,i} - Q_{dmin,i}} & \text{if } Q_{dmin,i} \leq q_{d,i} \leq Q_{d,i} \end{cases} \quad [25]$$

With the above definition, the action space is now bounded between -1 and 1, with optimal actions tending to 0 as the drug concentration approaches steady state at the outlet.

Two drug control with the deterministic model

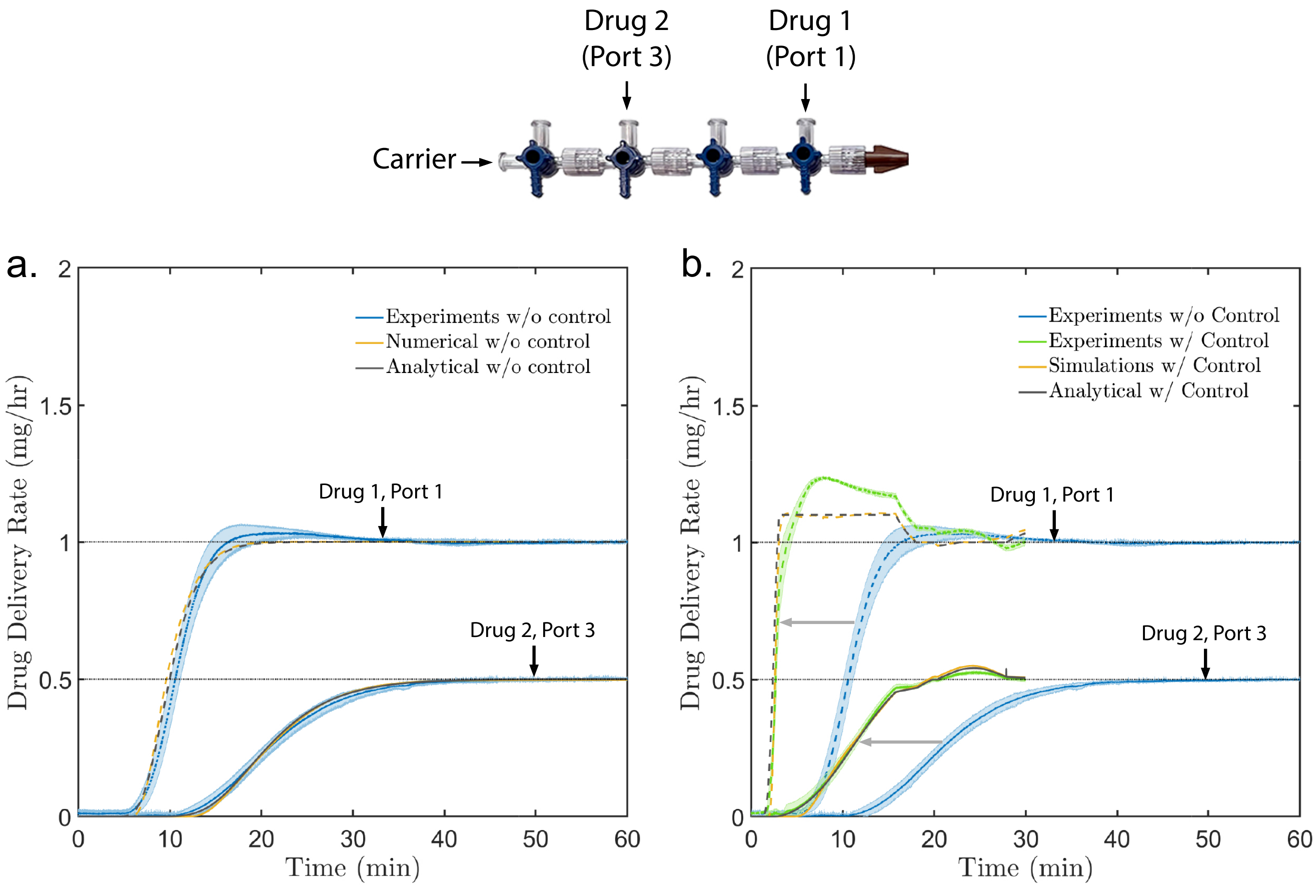

Fig. S10. Two drug infusion without control **a.**, and with control using the deterministic model **b.**.

Reinforcement Learning Model

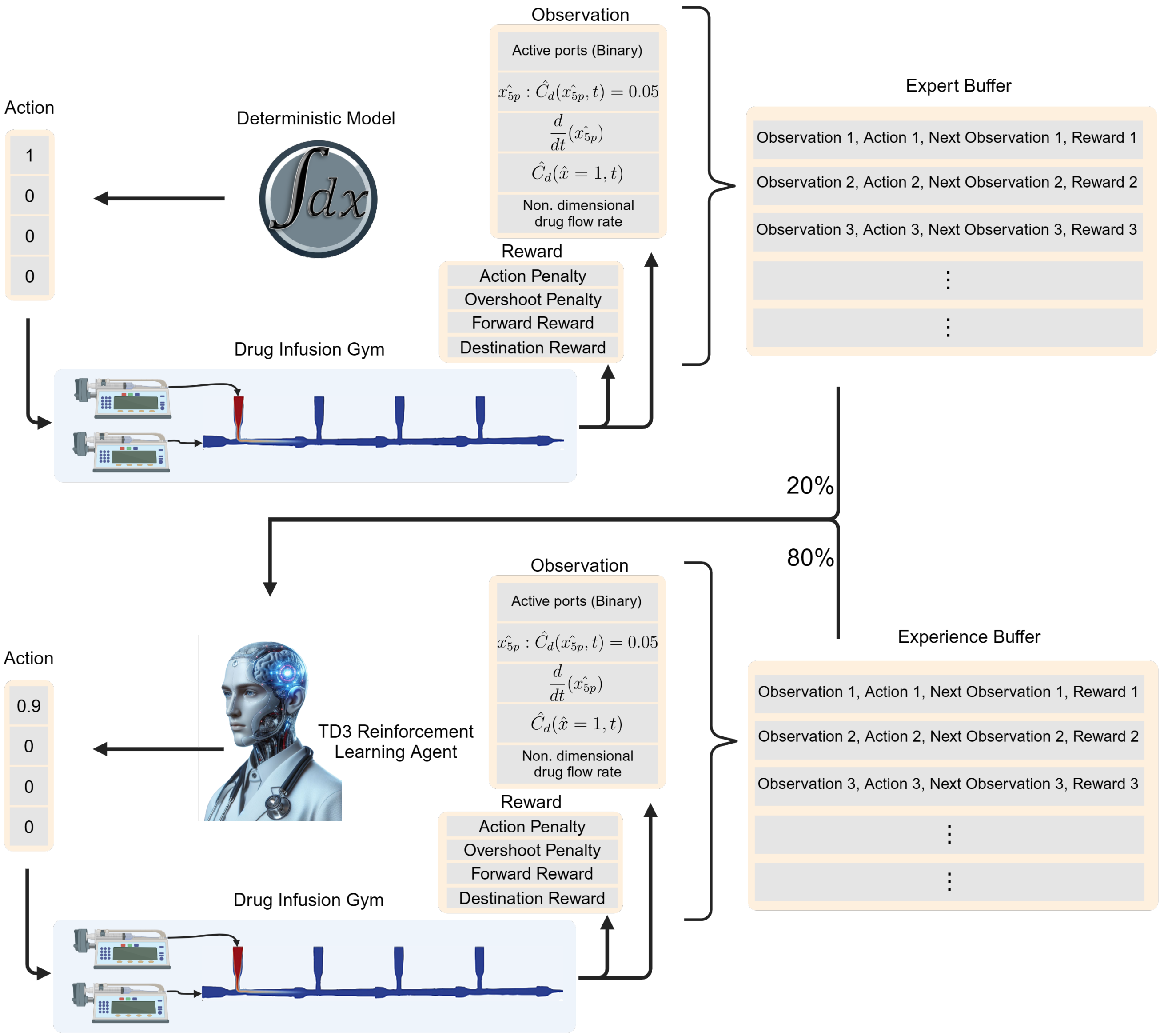

Fig. S11. a. Training methodology for the TD3 reinforcement learning agent with expert training via a 20-80 split buffer sampling.

Model architecture. The reinforcement agent has an actor-critic architecture with each network having 72960 neurons. The agent has a split buffer, which is partly populated by expert trajectories obtained from the deterministic model reported in main text section (*Deterministic policy for rapid drug delivery*). This buffer is sampled with a ratio of 20:80 (expert:standard training buffer) with a total batch size of 2560. The exact implementation and hyperparameters used are available in GitHub.

Observations. The agent receives six types of observations for each of the four ports:

- Active ports: A binary string with a value of 1 for each port having an active drug, 0 otherwise.
- $\hat{x_{5p}} : \hat{C}_d\left(\hat{x_{5p}}, t\right) = 0.05$: The non-dimensional location along the catheter assembly where the drug concentration is 5% of its set value. The non-dimensional location is 0 at port 4 and 1 at the tip of the catheter.
- $\frac{d}{dt}\left(\hat{x_{5p}}\right)$: The velocity of $\hat{x_{5p}}$.
- $\hat{C}_d\left(1, t\right)$: The non-dimensional concentration at the tip of the catheter.
- Non-dimensional drug flow rate: $\hat{C}_d\left(1, t\right) * \left(q_c + \Sigma q_{d,i}\right) / \left(Q_c + \Sigma Q_{d,i}\right)$

- Flow rate factor: $Q_{dmax,i}/Q_{d,i}$.

Rewards. The agent receives four types of rewards based on its actions:

- Action Penalty: This is a two component negative reward that penalizes large changes in actions as well as actions on ports without drugs. Implemented as below,

```
    actionPenalty = -np.sum(abs(self.prevAction - action) * penaltyFactor) / self.numPorts
                    - offTargetPenaltyFactor * np.sum(abs((1 - self.activeDrugPorts) * action))
```

- Overshoot Penalty: Penalizes the agent when the drug delivery overshoots beyond 50% of the set delivery rate. Implemented as below,

```
    overshootPenalty = - np.sum(currentNonDimDrugFlowRate[currentNonDimDrugFlowRate > 1.5])
```

- Forward Reward: Rewards the agent when the drug delivery rate is made to approach the set rate. Implemented as below,

```
    forwardReward = -abs(1.0 - np.mean(currentNonDimDrugFlowRate))
```

- Destination Reward: Rewards the agent when the both the drug flow rates and pump infusion rates reach the set value. Implemented as below,

```
    destinationReward = 1000
```

Deterministic guardrails. The action space is constrained between $[-1, 1]$, where the flow rates are non-dimensionalized according to Eqs.24 and 25. As a result the model is hardwired to respect constraints given by Eq.19 and 20. The remaining constraints are softwired in the model through the rewards specified above. To ensure that the model does violate these constraints (Eq. 21, 22 and 23), we have a simple wrapper that limits the model actions to stay within the system constraints.

Validation reward with and without deterministic trajectories

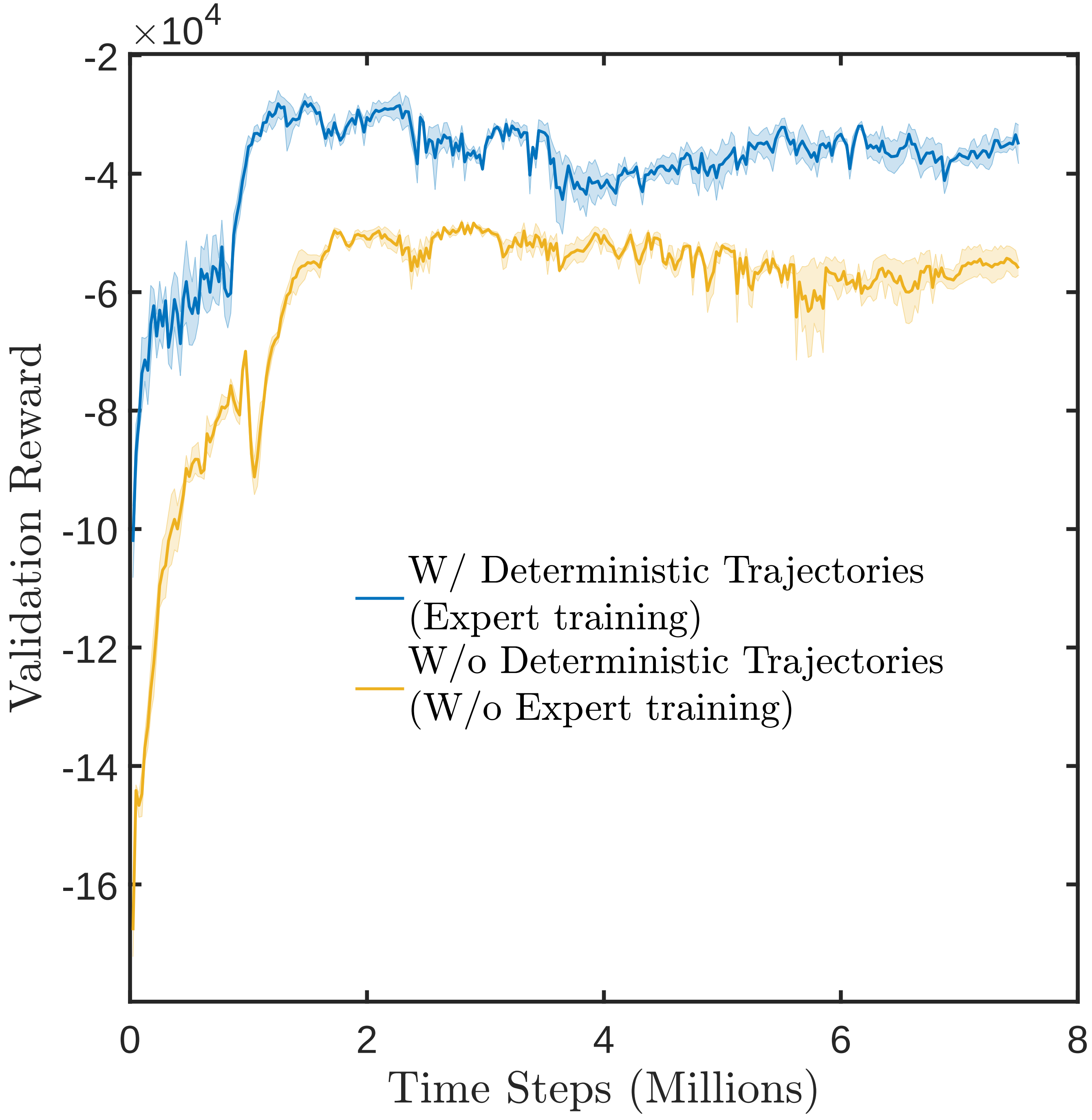

Fig. S12. a. Expert training improves both the rate of learning and the ultimate performance of the RL agent

Reinforcement learning single drug control

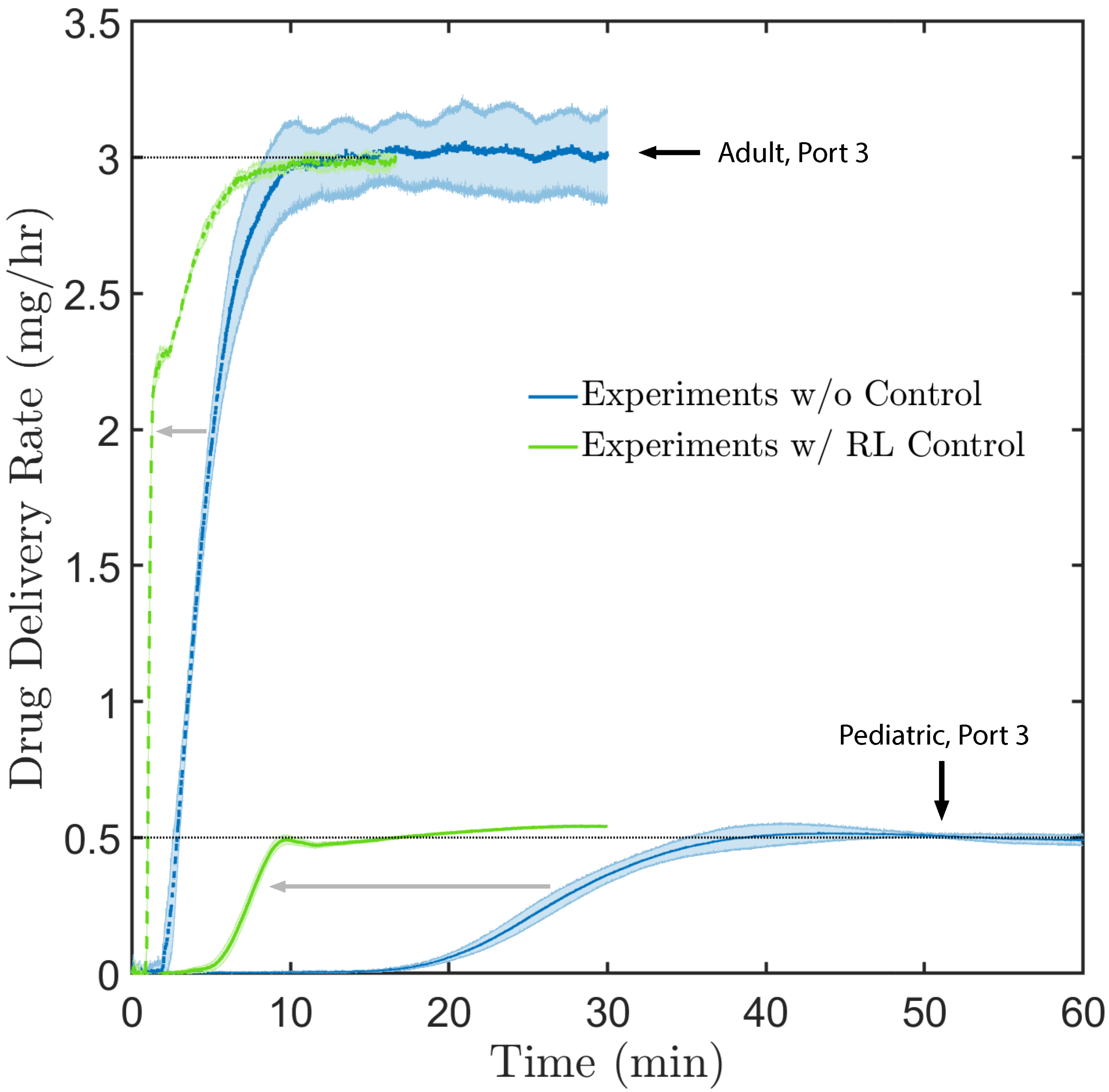

Fig. S13. a. Single drug control with reinforcement learning.

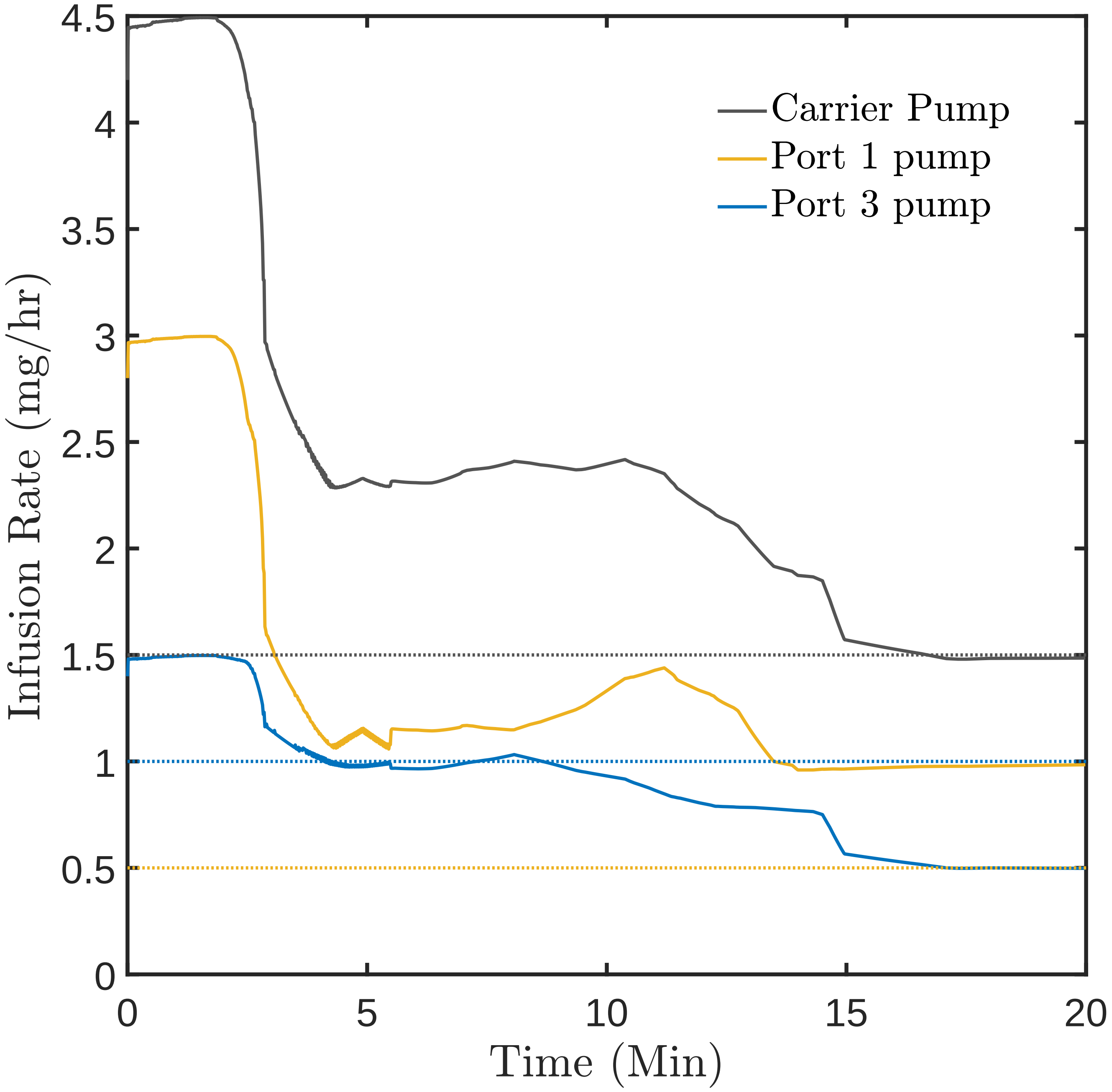

Fig. S14. a. Control trajectories executed by the RL for two drug infusions.

Performance of Raspberry Pi

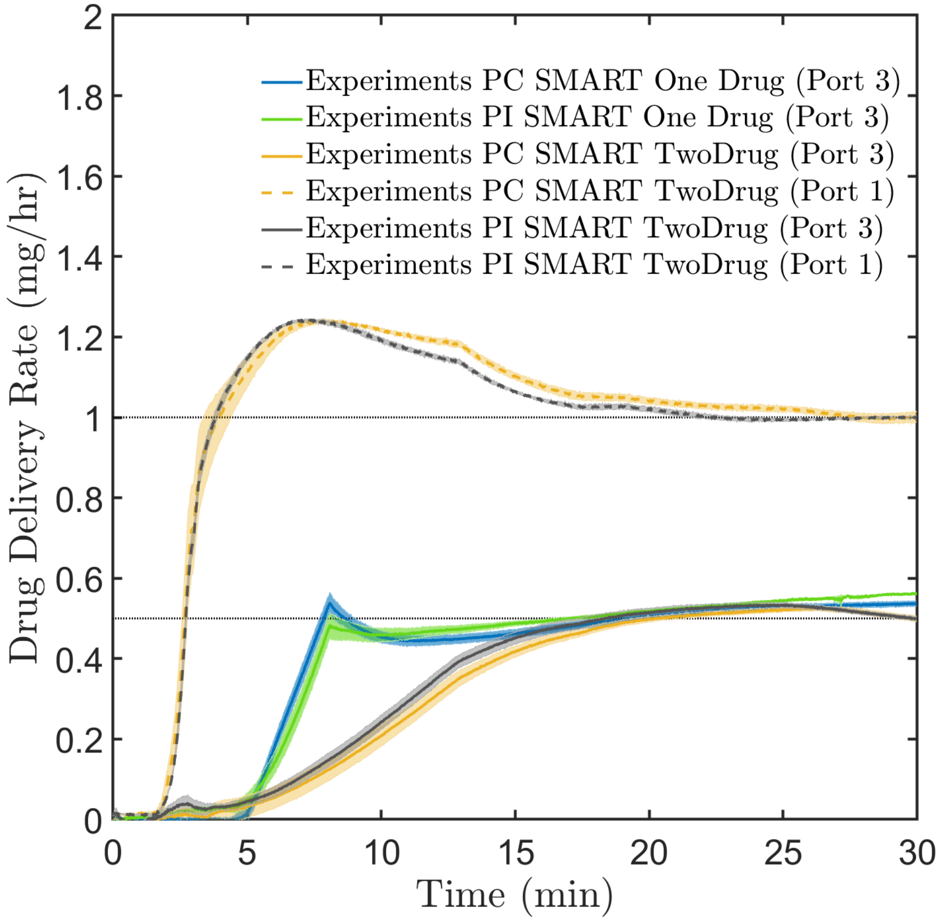

Fig. S15. The developed algorithms are optimized to run faithfully on a single board computer (Raspberry Pi denoted by PI) with no statistical performance difference as a compared to a high performance workstation (denoted by PC).

Flush valve

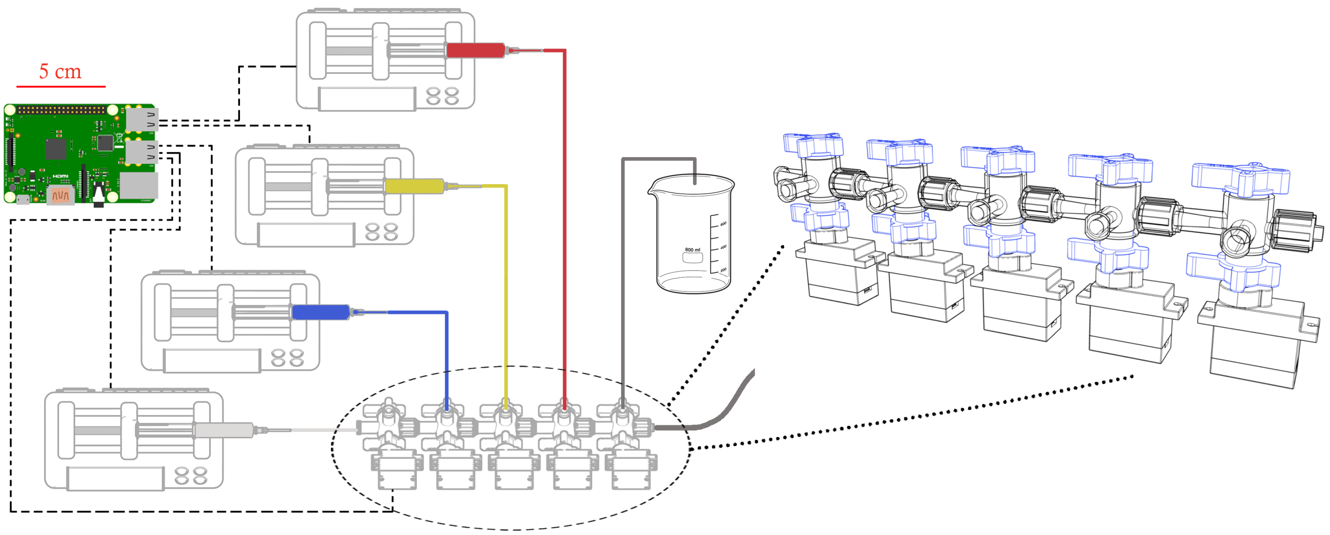

Fig. S16. Schematic of a servo-controlled stopcock assembly integrated with infusion pumps controlled by a single-board computer running SMART

Fig S16 displays a stopcock assembly made up of five commercially available stopcocks, each controlled by a servomotor (Servo Motor MG995 360°) capable of independent actuation. As the processor for controlling the servomotors, an Arduino Uno Rev3 is connected to a power supply and programmed with a code that can read commands sent via serial from the main computer or a single-board computer (Raspberry PI) and translate them into commands for each of the servomotors. An example of how the computer sends a command through the serial port is: In this example, the four servos (A, B, C, and D) will be positioned at 180°, 90°, 0°, and 270° respectively.

The flush valve is the last stopcock in the assembly and is connected to an empty reservoir that is used to completely empty the fluid within the stopcock assembly when necessary.

Impact of infusion mechanism

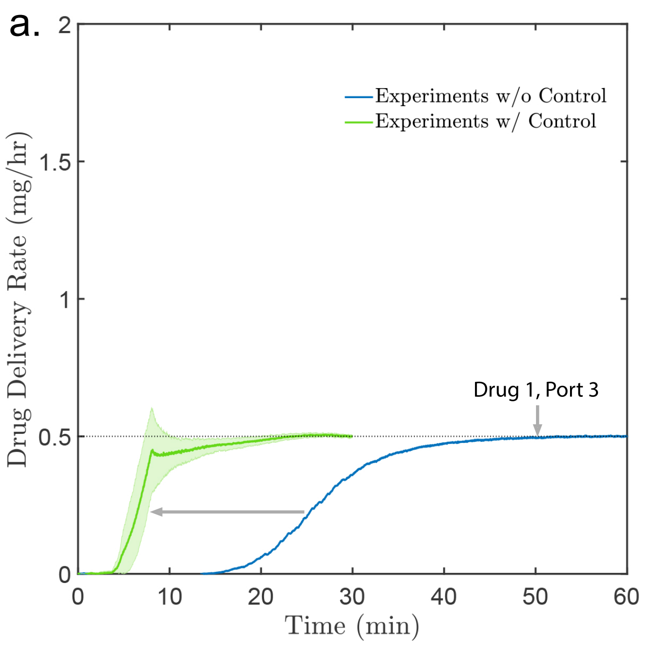

Fig. S17. a. Single drug control with peristaltic pumps. b. Two drug control with peristaltic pumps.

References

1. Oualha M, et al. (2014) Population pharmacokinetics and haemodynamic effects of norepinephrine in hypotensive critically ill children. *British journal of clinical pharmacology* 78(4):886–897.
2. Beloeil H, Mazoit JX, Benhamou D, Duranteau J (2005) Norepinephrine kinetics and dynamics in septic shock and trauma patients. *British journal of anaesthesia* 95(6):782–788.
3. Eisele D, Smith RV (2008) *Complications in Head and Neck Surgery E-Book*. (Elsevier Health Sciences).
4. Salvadore G, Singh JB (2013) Ketamine as a fast acting antidepressant: current knowledge and open questions. *CNS neuroscience & therapeutics* 19(6):428–436.
5. Smith MD, Maani CV (2023) Norepinephrine in *StatPearls [Internet]*. (StatPearls Publishing).
6. Lee S (2019) Dexmedetomidine: present and future directions. *Korean journal of anesthesiology* 72(4):323–330.
7. Bondi DS, Ohler KH (2017) Vasopressin and hemodynamic effects on the neonate. *NeoReviews* 18(8):e460–e471.
8. Twiner MJ, Hennessy J, Wein R, Levy PD (2022) Nitroglycerin use in the emergency department: current perspectives. *Open Access Emergency Medicine* pp. 327–333.
9. Marik PE, Varon J (2009) Perioperative hypertension: a review of current and emerging therapeutic agents. *Journal of clinical anesthesia* 21(3):220–229.
10. Nguyen LP, Gerstein NS (2019) Chapter 11 - cardiovascular pharmacology in noncardiac surgery in *Essentials of Cardiac Anesthesia for Noncardiac Surgery*, eds. Kaplan JA, Cronin B, Maus TM. (Elsevier, New York), pp. 247–288.
11. Richards E, Lopez MJ, Maani CV (2024) Phenylephrine.
12. Folino TB, Muco E, Safadi AO, Parks LJ (2022) Propofol in *StatPearls [Internet]*. (StatPearls Publishing).
13. Sheth S, Holtsman M, Mahajan G (2018) Major opioids in pain management in *Essentials of pain medicine*. (Elsevier), pp. 373–384.
14. Davidov M, Kakaviatos N, Finnerty JR FA (1967) Antihypertensive properties of furosemide. *Circulation* 36(1):125–135.
15. Giardina EG, Passman R (year?) Amiodarone: Clinical uses (https://www.uptodate.com/contents/amiodarone-clinical-uses). Accessed: 2024-08-31.
16. Bakhsh A, et al. (2021) Immediate intravenous epinephrine versus early intravenous epinephrine for in-hospital cardiopulmonary arrest. *BMC anesthesiology* 21(1):147.
17. Gill W, Sankarasubramanian R (1970) Exact analysis of unsteady convective diffusion. *Proceedings of the Royal Society of London. A. Mathematical and Physical Sciences* 316(1526):341–350.
