# Supplementary figures and images for: Transport physics‐informed reinforcement learning agents deployed in standalone infusion pumps for managing multidrug delivery in critical care
Source: Bioeng Transl Med. 2025 Mar 18;10(5):e70013. doi: 10.1002/btm2.70013 (PMC12478332; doi:10.1002/btm2.70013)

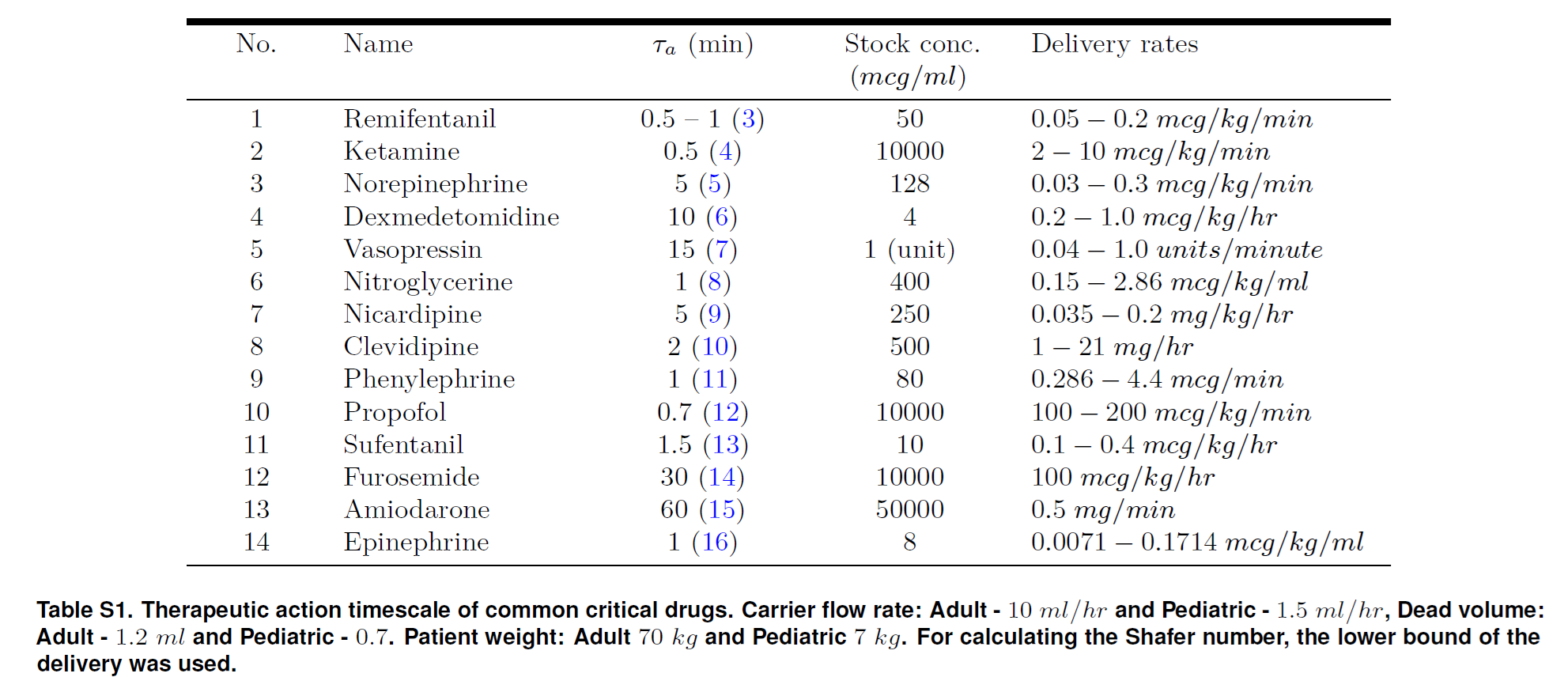

Supplement: Supplementary file 2 — TABLE S1. Therapeutic action timescale of common critical drugs. Carrier flow rate: adult—10 mL/h and pediatric—1.5 mL/h, dead volume: adult—1.2 mL and pediatric—0.7. Patient weight: adult—70 kg and pediatric—7 kg. For calculating the Shafer number, the lower bound of the delivery was used. [file BTM2-10-e70013-s001.png]
